# Human leukemia cells (HL-60) proteomic and biological signatures underpinning cryo-damage are differentially modulated by novel cryo-additives

**DOI:** 10.1093/gigascience/giy155

**Published:** 2018-12-10

**Authors:** Noha A S Al-Otaibi, Juliana S Cassoli, Daniel Martins-de-Souza, Nigel K H Slater, Hassan Rahmoune

**Affiliations:** 1 Department of Chemical Engineering & Biotechnology, University of Cambridge, Philippa Fawcett Drive, Cambridge CB3 0AS, United Kingdom; 2 King Abdulaziz City for Science and Technology, Kingdom of Saudi Arabia, P.O Box 6086, Riyadh 11442, Saudi Arabia; 3 Laboratory of Neuroproteomics, Department of Biochemistry and Tissue Biology, Institute of Biology, University of Campinas (UNICAMP), Campinas, SP, Brazil

**Keywords:** cryopreservation, oxidative stress, dimethylsulfoxide, nigerose, salidroside

## Abstract

**Background:**

Cryopreservation is a routinely used methodology for prolonged storage of viable cells. The use of cryo-protective agents (CPAs) such as dimethylsulfoxide (DMSO), glycerol, or trehalose is paramount to reducing cellular cryo-injury, but their effectiveness is still limited. The current study focuses on establishing and modulating the proteomic and the corresponding biological profiles associated with the cryo-injury of human leukemia (HL-60) cells cryopreserved in DMSO alone or DMSO +/- novel CPAs (e.g., nigerose [Nig] or salidroside [Sal]).

**Findings:**

To reduce cryo-damage, HL-60 cells were cultured prior and post cryopreservation in malondialdehyde Roswell Park Memorial Institute medium-1640 media +/- Nig or Sal. Shotgun proteomic analysis showed significant alterations in the levels of proteins in cells cryopreserved in Nig or Sal compared to DMSO. Nig mostly affected cellular metabolism and energy pathways, whereas Sal increased the levels of proteins associated with DNA repair/duplication, RNA transcription, and cell proliferation. Validation testing showed that the proteome profile associated with Sal was correlated with a 2.8-fold increase in cell proliferative rate. At the functional level, both Nig and Sal increased glutathione reductase (0.0012±6.19E-05 and 0.0016±3.04E-05 mU/mL, respectively) compared to DMSO controls (0.0003±3.7E-05 mU/mL) and reduced cytotoxicity by decreasing lactate dehydrogenase activities (from -2.5 to -4.75 fold) and lipid oxidation (-1.6 fold). In contrast, only Nig attenuated protein carbonylation or oxidation.

**Conclusions:**

We have identified key molecules and corresponding functional pathways underpinning the effect of cryopreservation (+/- CPAs) of HL-60 cells. We also validated the proteomic findings by identifying the corresponding biological profiles associated with promoting an anti-oxidative environment post cryopreservation. Nig or Sal in comparison to DMSO showed differential or additive effects in regard to reducing cryo-injury and enhancing cell survival/proliferation post thaw. These results can provide useful insight to cryo-damage and the design of enhanced cryomedia formulation.

## Background

Cryopreservation of viable cells and tissues is a powerful approach to ensure cell longevity and integrity and facilitate cell/tissue engineering therapy [1]. Cell-based therapy is a rapidly emerging industry and is estimated to be worth around $5 billion in the United States alone [2]. Despite well-established cryopreservation protocols, cells remain subject to a high level of cryo-damage, leading to compromised cell function and necrosis [3]. The cellular damage is generally seen as lipid and protein oxidation, which can severely affect cell stability [4] and its ability to proliferate [5]. Thus, reducing the impact of cryo-damage is paramount to enhance the cell recovery rate post freeze/thaw cycles.

Despite their reported toxic properties, dimethylsulfoxide (DMSO) and glycerol are the most commonly used cryo-protective agents (CPAs) to reduce cryo-injury and increase cell viability [5]. Other CPAs such as trehalose have been used for their cryo-protective properties against intracellular ice crystal formation [6]. However, the protective effect of these compounds is still limited [7] with low cell viability and recovery rates post cryopreservation [8]. The use of CPAs can also lead to production of reactive oxygen species, whereby cells are subjected to oxidative damage during freeze-thaw cycles [9]. Moreover, the effectiveness of intracellular or auto anti-oxidative response to cryo-insult is limited as cell survival is reduced [10]. Attempts to promote cellular anti-oxidative status have been reported before, and these showed an improved cell survival rate [11]. For example, the use of arabidopsis thaliana containing high levels of ascorbic acid increased intracellular catalase activity leading to a higher cell survival rate post thaw [11].

The majority of studies on cryopreservation have focused on either fertility [12–14] or more recently on stem cells [5]. The potential clinical use of human mesenchymal stem cells in regenerative medicine and/or cell-based therapy has led to a sharp focus on enhancing the cryopreservation process of these cells. Martín-Ibáñez et al. have succinctly summarized the current use of CPAs as additive (e.g., DMSO/glycerol +/- cryo-additive agents) to slightly improve the cryopreservation of human pluripotent stem cells [15]. More recently, Gurruchaga et al. have demonstrated that the combination of CPAs such as DMSO/sucrose has significantly improved the quality of human mesenchymal stem cells post cryopreservation [16]. Tissue cryopreservation of the umbilical cord has also been attempted, which is crucial to the future success of regenerative medicine [17]. Limited attempts have been carried out to improve cryopreservation of cell lines (e.g., hepatocytes) [18]. Moreover, the bulk of empirical studies attempting to decipher molecular profiles associated with cryo-injury have been conducted mainly on fertility-related specimens [19, 20], plant cells [21], or stem cells [22]. Likewise, attempts to modify cryo-proteomic profiles using CPAs or DMSO +/- antifreeze have been made mainly in the field of reproductive medicine [23, 24]. In contrast, only a limited number of molecular/functional studies have been conducted on nucleated human cell lines to decipher and modulate biological pathways underpinning cryo-damage.

Here, we have used human leukemia (HL-60) cells as a nucleated cellular model to establish the biomolecular profiles associated with cryo-damage in the presence of DMSO alone or with the addition of salidroside (Sal) or the novel CPA nigerose (Nig) [4]. The addition of Sal with the tyrosol glucoside, as the active component of the herb *Rhodiolarosea*, was used previously to prevent high-altitude sickness [25]. Sal has also been found to act as an antioxidant against hydrogen peroxide-induced apoptosis of human red blood cells [26] and as a CPA for red blood cell cryopreservation [4]. However, this is the first investigation to test the potential cryoprotective properties of Nig. Nig is an un-fermentable sugar obtained by partial hydrolysis of nigeran and is polyol extracted from fermentation of microorganisms such as black mold or dextrans [27] as well as honey [28]. A hypothesis-driven approach is clearly needed here to elucidate and modify cell-specific molecular and biological pathways associated with cryo-injury. Here, we have employed a shotgun proteomics approach to profile and modulate the molecular pathways underpinning human nucleated cell cryo-damage. The present study also offers the opportunity to enhance future cryomedia formulation, minimize losses of cell viability, and maximize cell recovery post freeze-thaw cycle.

## Data Description

For proteomic analysis, samples were analyzed using high-resolution mass spectrometry on a Synapt G2-Si HDMS mass spectrometer (Waters). Data processing, database searches, and label-free quantification were performed using Progenesis QI for Proteomics. The mass spectrometry raw data files, database search, and quantification results have been deposited and can be accessed via ProteomeXchange [29] with identifier PXD006998. The resulting HL-60 proteome profiles led us to investigate the corresponding biological activities (e.g., enzymatic, protein/lipid oxidation, and cell proliferation assays) post cryopreservation.

### Analyses

**Figure 1:**
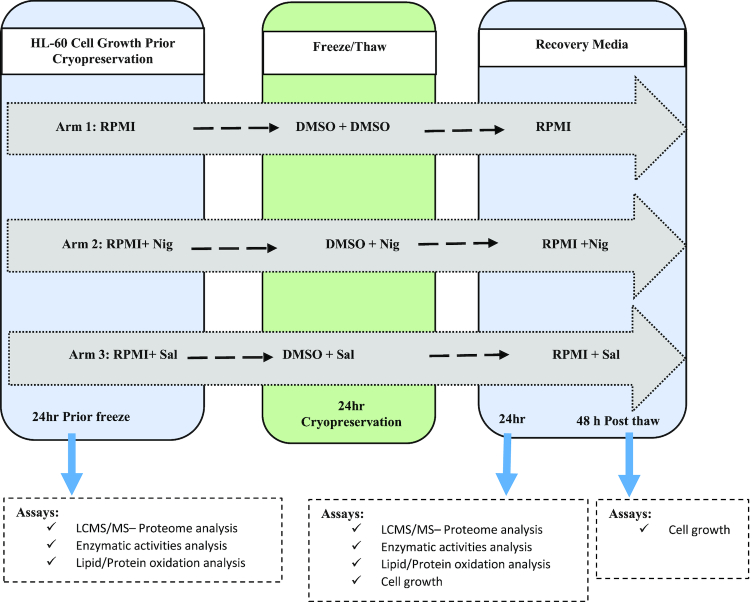
Schematic diagram. Experimental design of HL-60 cryopreserved in dimethylsulfoxide (DMSO) [n = 5] +/- Nigerose (Nig) [n = 5 replicates] or Salidroside (Sal) [n = 5 replicates]. Proteomic analysis and corresponding biological assays were conducted 24 hours prior and post cryopreservation of HL-60 cell cultures grown in Roswell Park Memorial Institute medium (RPMI)-1640 media +/- Nig or Sal.

**Figure 2:**
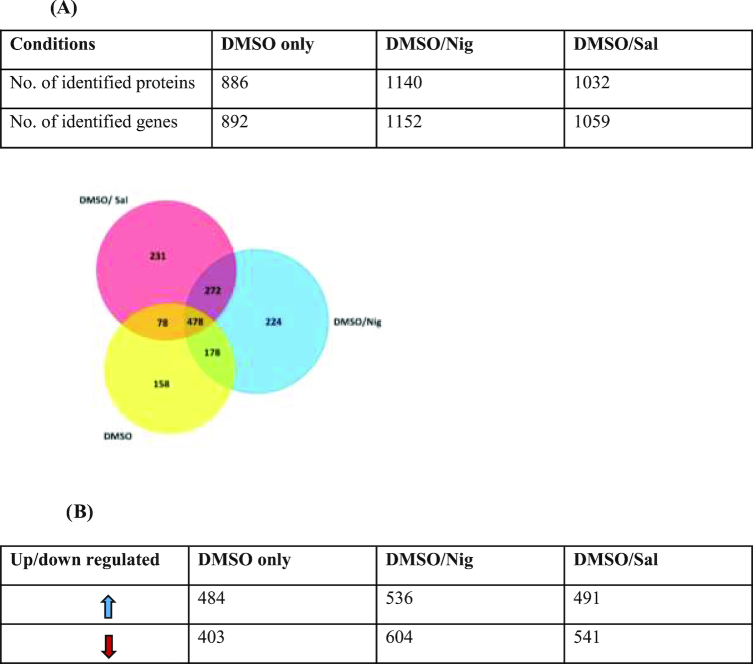
Proteome analysis. HL-60 total number of differentially expressed proteins cryopreserved in DMSO +/- Nig or Sal (n = 5 per arm). **(A)** Venn diagram illustrating HL-60 cells unique and overlapped number of significantly changing proteins 24 hours prior and post thaw. The numbers in the circles represent the number of identified genes significantly changing prior/post HL-60 cryopreserved in DMSO only (n = 5 replicates), DMSO + Nig (n = 5 replicates), or DMSO + Sal (n = 5 replicates). **(B)** Table representing the total number of identified genes representing HL-60 upregulated (blue arrow) and downregulated (red arrow) proteins in each of the above cryo-condition.

Proteins found to present at significantly different levels, between the different arms of the study (Fig. 1), in HL-60 cells cryopreserved in DMSO alone (n = 5 replicates), DMSO+Nig (n = 5 replicates), or DMSO+Sal (n = 5 replicates) were classified according to their biological and functional pathways. The Uniprot accession codes of differentially expressed proteins or genes were mapped to Gene Ontology Annotation using software linked to Funrich database [29-31]. The number of significantly changing proteins (*P* < 0.05) that are expressed in HL-60 cryopreserved in DMSO, DMSO + Nig, or DMSO + Sal are illustrated in a Venn diagram (Fig. 2A). Thus, the overlapping as well as the uniquely expressed proteins (e.g., up/down-regulated) are shown in (Fig. 2A).

### Proteomic analyses

Label-free quantitative shotgun proteomic analysis was used to identify HL-60 cell proteins found at different levels post cryopreservation in DMSO alone, DMSO +Nig, or DMSO + Sal (n = 5 replicates/arm). In this study, cryopreservation has significantly induced changes in the abundance of many proteins of HL-60 cryopreserved in the DMSO +Nig group (1,140 proteins; Supplementary Table S2) and the DMSO + Sal group (1,032 proteins; Supplementary Table S3), with only 886 proteins found changing for HL-60 cryopreserved DMSO alone (Supplementary Table S1). Some of the biologically relevant proteins expressed by HL-60 (i.e., identified, quantified, and differentially expressed) are summarized in Table 1.

**Table 1:** Proteins found at significantly different levels (*P* < 0.05) using label-free liquid chromatography-high resolution mass spectrometry/mass spectrometry (LCMS/MS) profiling of the human promyelocytic leukemia HL-60 cells cryopreserved in DMSO (n = 5 replicates), +/- Sal (n = 5 replicates), or Nig (n = 5 replicates)

		DMSO alone		DMSO/nigerose		DMSO/salidroside	
Uniprot entry	Protein name	UP	FC(log2PC/PT)	UP	FC (log2PC/PT)	UP	FC (log2PC/PT)
	**Oxido-Redox**						
Q99497	Protein deglycase DJ-1	ND		ND		12 1.4	
P00338	Lactate dehydrogenase A chain	11	-1.6	ND		11	-1.6
P00390	Glutathione reductase	7	3.2	ND		ND	
P00441	Superoxide dismutase [Cu-Zn]	8	1.4	ND		ND	
Q16881	Thioredoxin reductase 1	2	14.6	2	35.0	2	15
P28331	Nicotinamide adenine dinucleotide (NADH)-ubiquinone oxidoreductase 75 kDa subunit	4	4.9	4	46.0	4	16
Q9Y2Q3	Glutathione S-transferase kappa 1	2	-8.0	2	-13.6	2	-3.5
P30048	Thioredoxin-dependent peroxide reductase, mitochondrial	2	-3.0	2	-5.2	2	-8.8
C9J0G0	Acyl-coenzyme A oxidase (ACOX)	2	32.0	2	16.8	2	42.7
P49748	Very long-chain specific acyl-CoA dehydrogenase	5	-2.7	5	-11.6	5	-14.8
P16152	Carbonyl reductase	ND		ND		5	-1.5
P49368	T-complex protein 1 subunit gamma	ND		17	1.2	ND	
P40227	T-complex protein 1 subunit zeta	ND		7	1.4	ND	
Q9NZL4	Hsp70-binding protein 1	3	14.4	3	-71	3	-77.0
P48723	Heat shock 70 kDa protein 13	ND		2	15.8	ND	
P34932	Heat shock 70 kDa protein 4	ND		ND		17	1.3
Q53EL6	Programmed cell death protein 4	ND		ND		4	1.6
P08758	Annexin A5 (Annexin-V)	6	-6.6	6	-9.2	6	4.5
Q5VT06	Centrosome-associated protein 350	29	88.9	29	61.2	29 81.2	
P25787	Proteasome subunit alpha type-2 (PSAT2)	ND		3	34.4	ND	
	**Nuclear activities regulation**						
Q9BTE3	Mini-chromosome maintenance complex-binding protein	ND		2	11.0	2	70.0
P33993	DNA replication licensing factor MCM7	ND		9 -3.5		9	- 2.4
P35658	Nuclear pore complex protein Nup214	ND		ND		6	1.6
Q86YP4	Transcriptional repressor p66-alpha	ND		ND		11	2.5
Q5T890	DNA excision repair protein ERCC-6-like	4	-14.4	4	-13.8	4	-8.8
Q99973	Telomerase protein component 1	ND		3	-2.3	3	-2.3
Q8WXI9	Transcriptional repressor p66-beta	4	-2.6	ND		ND	
O14980	Exportin-1	5	3.0	ND		5	3.7
A6H8Y1	Transcription factor TFIIIB component B	9	2.1	9	7.9	9	10.6
Q15054	DNA polymerase delta subunit 3	2	- 3.4	2	- 30.0	2	-22.3
	**Cell growth and function**						
P00533	Epidermal growth factor receptor	ND		ND		4	- 2.1
Q14676	Mediator of DNA damage checkpoint protein 1	ND		5	17.0	5	21.4
Q6ZUM4	Rho GTPase-activating protein 27	2	13.7	2	39.5	2	75.4
Q9BYX2	TBC1 domain family member 2A	3 10.5		3	11.2	3	39.0
O14976	Cyclin-G-associated kinase	4	4.1	4	8.5	4	9.8
Q8N163	Cell cycle and apoptosis regulator protein 2	9 1.5		9	1.8	9	2.3
O94986	Centrosomal protein 152 KDa	ND		7	59.8	7	19.0
Q13576	RasGTPase-activating-like protein IQGAP2	4	15.2	4	65.7	4	40.9
Q14789	Golgin subfamily B member	14	18.9	14	37.2	14	21.3
P49327	Fatty acid synthase	ND		39	10.4	39	9.0
Q01484	Ankyrin-2	17	32.0	17	39.7	17	48.8
O00423	Echinoderm microtubule-associated protein-like 1	4	23.0	4	42.0	4	32.2
A0A0U1RR07	Synaptotagmin-like protein 2	4	4.1	4	9.0	4	22.0
Q15691	Microtubule-associated protein RP/EB family member 1	10	7.1	10	3.2	10	7.1
E9PNZ4	Microtubule-actin cross-linking factor 1, isoforms 1/2/3/5	2	12.6	2	12.3	2	4.4

Abbreviations: UP = unique peptides; ND = not detected; FC = fold changes indicating the ratio of differentially expressed proteins identified prior cryopreservation (PC) and post thaw (PT).

**Figure 3:**
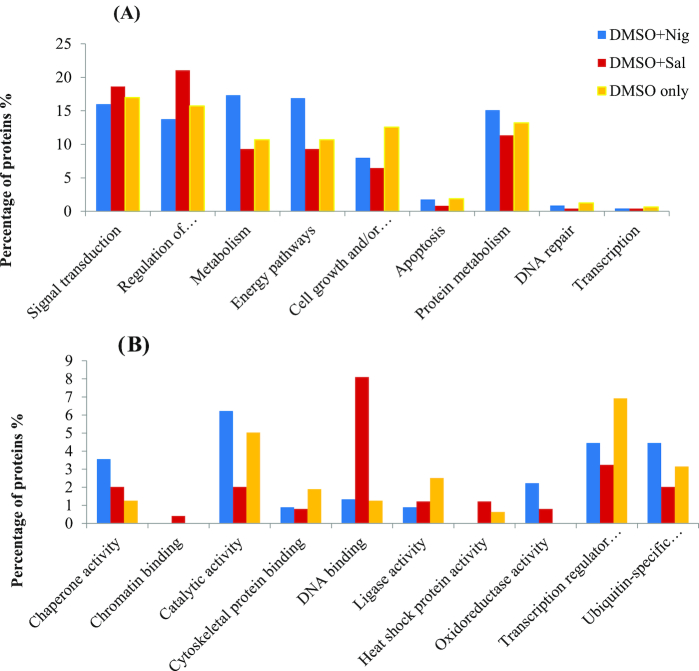
Biological pathways analysis. Comparative overview of the biological processes **(A)** and functional functions **(B)** representing mammalian HL-60 cells cryopreserved in DMSO +/- Nig or Sal. The percentage of proteins extracted from HL-60 cells cryopreserved in DMSO alone, DMSO/Nig, or DMSO/Sal were identified using FunRich software.

Using the Funrich database, the *in silico* functional analysis of the proteomes has revealed the following:

- The effect of cryopreservation showed a higher number of differentially expressed 1,140 proteins (with *P* < 0.05) for DMSO + Nig (Fig. 2A) and DMSO + Sal (1,032 proteins; Fig. 2A), with only 886 proteins found for DMSO alone (Fig. 2A). In addition, the Venn diagram analysis (Fig. 2A) has shown that the largest number of uniquely identified proteins was found in DMSO + Sal (n = 231). Cells cryopreserved DMSO + Nig showed 224 proteins that are specifically expressed in the presence of Nig, while the lowest number (n = 158) of uniquely expressed proteins (not found in DMSO + Sal or Nig treated cells) is in HL-60 cells cryopreserved in DMSO.
- The nature of biological pathways associated with cryo-damage of HL-60 cryopreserved in DMSO alone and those that were differentially modulated by the CPAs post thaw. A proportionately high number of proteins (21.05%) engaged in nucleotide and nucleobase regulation or DNA binding were identified in HL-60 cells cryopreserved in DMSO + Sal. In contrast, the DMSO + Nig arm showed the highest proportion of changes (16.8%) in proteins associated with energy pathways and protein metabolism (Fig. 3A). Supplementing DMSO with Nig or Sal as CPAs also led to an increased level of proteins with oxidoreductase activities, especially in the case of Nig (Fig. 3B). The level of proteins linked to cell maintenance was the highest in HL-60 cells cryopreserved in DMSO alone (12.5%) when compared to DMSO + Nig (8%) and DMSO + Sal (6.4%).
- The percentage of recognized DNA binding proteins was estimated at 8.09% for cells cryopreserved in DMSO + Sal, while this did not exceed 2% in cells cryopreserved in DMSO + Nig and DMSO alone (Fig. 3B). HL-60 protease activity-associated proteins were estimated at 4.4% in DMSO + Nig, 3.1% in DMSO alone, while only reaching 2.02% in DMSO + Sal (Fig. 3B). With regards to cryo-stress, heat shock proteins were differentially expressed in HL-60 cells cryopreserved in DMSO + Sal (1.2%) and DMSO alone (0.6%), whereas these proteins were not detected in cells cryopreserved in the presence of Nig. The proteome profile reflecting the effect of freeze/thaw cycle on HL-60 cells cryopreserved in DMSO alone and DMSO + Nig or Sal are summarized in Table 1.

### Oxido-redox functions

Reduction in HL-60 cryo-oxidation was shown by an increased level of glutathione reductase and superoxide dismutase [Cu-Zn] by 3.2 and 1.4 fold, respectively, in DMSO alone (Table 1). However, no significant change was detected in the levels of either of these markers for cells preserved in the presence of Nig or Sal. In contrast, the levels of thioredoxin reductase-1 were increased up to 35 fold when Nig was added and by 15 fold with the addition of Sal. A similar pattern was seen with the NADH-ubiquinone oxidoreductase 75 kDa subunit. The levels of pro-oxidative enzymes were all reduced in the presence of CPAs such as peroxiredoxin (not detected in DMSO, downregulated by 2.0 fold in DMSO + Nig and by 3.5 fold in DMSO + Sal), glutathione S-transferase Kappa-1 (decreased by 8 fold in DMSO, decreased 13.6 fold in DMSO + Nig, and decreased 3.5 fold in DMSO +Sal), and thioredoxin-dependent peroxide reductase (decreased 3.0 fold in DMSO, decreased 5.2 fold in DMSO + Nig, and decreased 8.8 fold in DMSO + Sal). Very long chain-specific acyl-CoA dehydrogenase (involved in fatty acid β-oxidation) showed a 4-fold decreased level in HL-60 cells cryopreserved in DMSO + Nig and a 5-fold decreased level in DMSO + Sal compared to the levels in cells cryopreserved in DMSO alone. A similar anti-oxidative pattern was observed in the presence of CPAs with increased levels of acyl-coenzyme A oxidase (16.8 fold in DMSO + Nig and 42.7-fold in DMSO + Sal) and carbonyl oxidase (5.5 fold in the presence of DMSO + Sal).

A differential response to cryo-stress was identified when Nig or Sal were added to media prior to and post cryopreservation of HL-60 cells. For example, the stress-related protein Hsp 70-binding protein 1 was increased 14.4-fold in HL-60 cells cryopreserved in DMSO alone, but its level decreased by 71 fold and 77 fold in the presence of Nig and Sal, respectively. In contrast, cytosolic stress response proteins such as the heat shock 70 kDa protein 4 was not detected in DMSO +/- Nig and was increased by 2.3 fold in DMSO + Sal. Finally, microsomal Hsp 70 protein-13 was not detected in HL-60 cells cryopreserved in DMSO +/- Sal, while this same protein was upregulated by 15.8 fold in the presence of DMSO + Nig.

### Nuclear and cellular functions

Twenty-four hours post thaw, incubation of HL-60 cells in Sal led to a marked elevation in its nuclear proteins as shown in Table 1. In the presence of Sal, the levels of proteins associated with DNA repair were relatively upregulated such as DNA excision repair protein ERCC-6-like (− 8.8-fold in Sal, while diminishing by 13.8 fold in the presence of DMSO + Nig and by 14.4 fold in DMSO alone), mini-chromosome maintenance complex-binding protein (increased by 71 fold in DMSO + Sal, 11 fold in DMSO +Nig, and not detected in DMSO alone). Sal also enhanced the levels of proteins involved in transcriptional regulation such as transcription factor TFIIIB component B protein (increased by 11 fold in DMSO + Sal, 8 fold in DMSO + Nig, and 2 fold in DMSO alone).

In the presence of CPAs, the significantly altered levels of proteins associated with nuclear activities were reflected by the changes in proteins associated with cell growth and cytosolic functions. For example, the presence of Sal and Nig doubled the fold change of cyclin-G-associated kinase from 4 fold in DMSO alone up to an 8- or 9-fold increment in Nig and Sal, respectively. TBC1 domain family member 2A, known to be involved in the regulation of GTPase activities and vesicle fusion, was only augmented by 10.5 fold post thaw for HL-60 cryopreserved in DMSO alone, while it was further enhanced in the presence of Nig by 11.2 fold and up to 39 fold increase in Sal. The levels of cytoskeletal proteins were also boosted by the CPAs such as ankyrin-2, microtubules-associated protein and echinoderm microtubule-associated protein-like, which are known to be associated with cell shape. Functions such as cell re-organization and division were also increased in the presence of Nig and Sal compared to DMSO alone (Table 1).

### HL-60 cell proliferation post thaw

**Figure 4:**
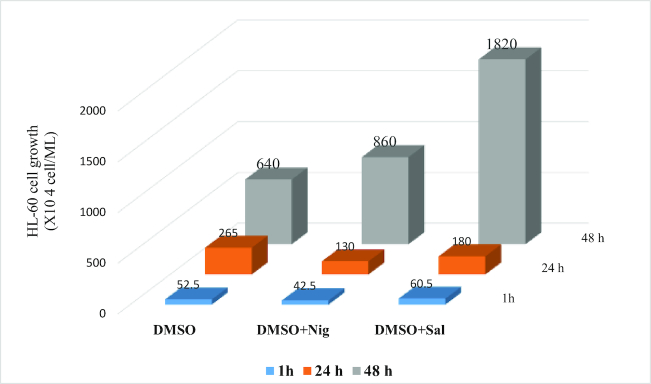
Cell growth. HL-60 cell proliferation was measured in duplicate at 1 hour, 24 hours, and 48 hours post thaw. Cells were initially either cultured in Roswell Park Memorial Institute medium (RPMI) media containing Nig (300 µM) or Sal (200 µM) and cryopreserved in DMSO +/- Nig or Sal. HL-60 cells were thawed, washed, and cultured in RPMI media containing Nig (300 µM) or Sal (200 µM) for up to 48 hours. Data are expressed as mean.

The number of HL-60 cells 24 hours post thaw was estimated at 265 × 10^4^, 130 × 10^4^, and 180 × 10^4^ cells/mL for DMSO alone, DMSO + Nig, and DMSO + Sal, respectively (Fig. 4). At 48 hours, Sal increased the proliferative rate by 2.84 fold compared to cells cryopreserved in DMSO alone, and this was 1.3 fold for DMSO + Nig compared to cells cryopreserved in DMSO alone (640 × 10^4^ cells/mL). The direct comparison between the effect of Nig and Sal on cell growth rate at 48 hours showed that the number of HL-60 cells in the presence Sal was at 1,820 × 10^4^ cells/mL, while this only reached 860 × 10^4^ cells/mL in the presence of Nig. Such an increase in the HL-60 cell proliferative rate post thaw in the presence of Sal was paralleled by the increase in the protein levels of cyclin-G-associated kinase (9.8 fold) (Table 1). Finally, post thaw HL-60 cells were immediately centrifuged and washed three times with culture media; the resulting changes in cell viability during the recovery period of up to 48 hours were negligible (<2%).

### Biological profiles of HL-60 cryopreserved in DMSO +/- Nig or Sal

HL-60 cell intracellular glutathione reductase (GR) activity was measured (n = 5 replicates) prior to freezing and 24 hours post thaw. GR activity was significantly increased in all cases. The presence of CPAs in the media significantly boosted GR activity from 0.0003 mU/mL prior to cryopreservation to 0.0005 mU/mL in the presence of DMSO alone. The addition of Nig boosted GR activity post thaw even further, reaching 0.0013±0.00006 mU/mL. Sal had the biggest effect on HL-60 cell GR activity with a reading of 0.0016 mU/mL (i.e., three times more increased compared to HL-60 cells cryopreserved in the standard DMSO cryomedia). HL-60 cell intracellular lactate dehydrogenase (LDH) activities were also measured prior to freezing and 24 hours post thaw (n = 5 replicates). Adding Sal to the culture or cryomedia lowered LDH readouts from 0.1±0.03 mU/mL in DMSO alone to 0.04±0.01 mU/mL in DMSO + Sal. Moreover, the addition of Nig had the biggest effect on lowering LDH activity by bringing this to 0.02±0.044 mU/mL (three times lower than prior to cryopreservation, five times less than DMSO alone, and two time less than DMSO + Sal).

**Figure 5:**
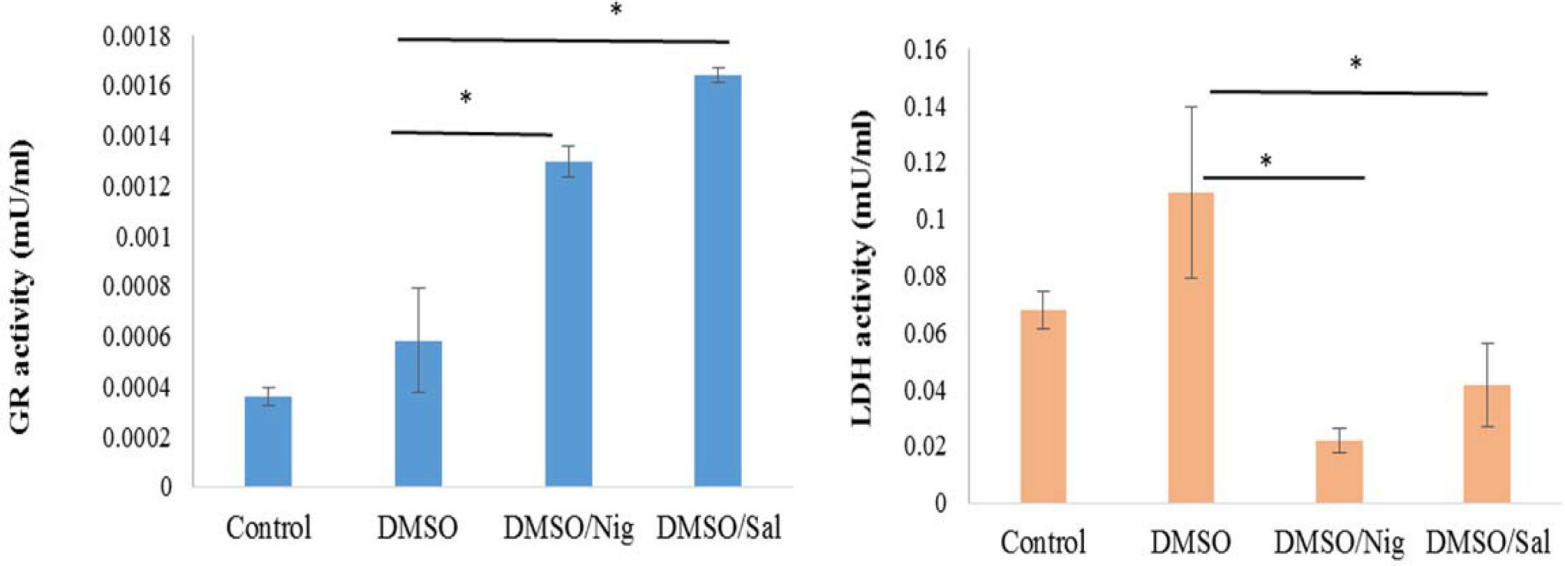
Oxido-redox enzymatic assays. Intra-cellular enzymatic activities of HL-60 were measured prior to freezing (control). Cells were frozen in DMSO +/- Sal or Nig and HL-60 GR and LDH activities were measured in Roswell Park Memorial Institute medium (RPMI) media only, RPMI + Nig (300 µM) or in RPMI + Sal (200 µM) 24 hours post thaw. **(A)** Glutathione reductase (GR) activity (mU/mL). **(B)** LDH activity (mU/mL). Data are presented as a mean (n = 5 replicates) ± standard deviation (*P value < 0.05).

Oxidation assays were conducted to investigate Nig and Sal cryo-protective properties against HL-60 cells lipid (e.g., lipid peroxidation) and protein (e.g., carbonylation) oxidation. HL-60 lipid peroxidation level was measured in triplicate prior to freezing and 1 hour and 24 hours post thaw in the presence and absence of Nig or Sal. Measurement of malondialdehyde (MDA) levels 1 hour post thaw showed a significant increase in lipid oxidation, with HL-60 cells cryopreserved in DMSO alone reaching an level of 7.31±0.16 nmol/mL (Fig. 5). In contrast, this was approximately 40% lower in the presence of Nig (4.35±0.02 nmol/mL) or Sal (4.53±0.09 nmol/mL). In the recovery phase (e.g., 24 hours post thaw), HL-60 cell lipid peroxidation levels reached control levels (e.g., prior to cryopreservation ∼2.1 nmol/mL). One day post thaw, lipid oxidation levels for HL-60cells cryopreserved in DMSO +/- Nig or Sal reversed back to their prior cryopreservation level (Fig. 5).

**Figure 6:**
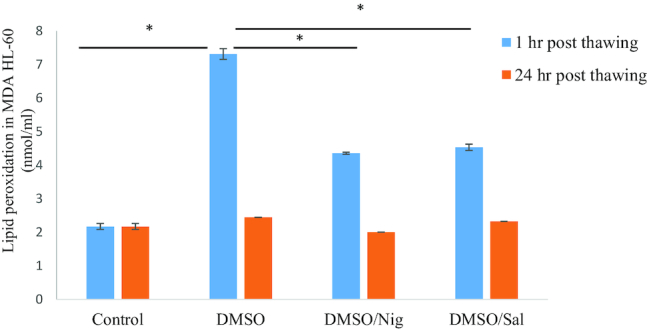
Lipid peroxidation (MDA) assay. Lipid oxidation of HL-60 incubated prior and post thaw in media +/- Nig or Sal and cryopreserved in DMSO +/- Nig (300 µM) or Sal (200 µM). The data are represented in mean (n = 3 replicates) ± standard deviation (*P value < 0.05).

**Figure 7:**
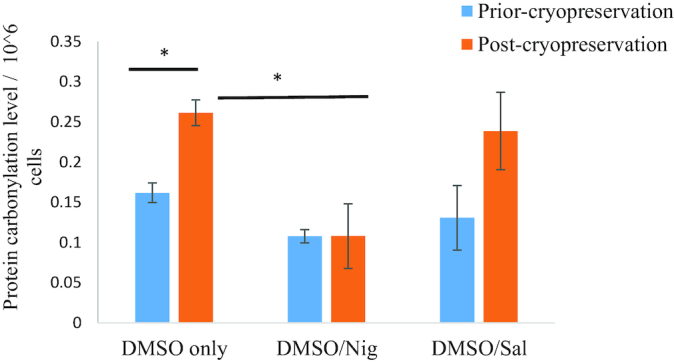
Protein carbonylation or oxidation of cryopreserved HL-60 cells. The control represents protein carbonylation level prior to HL-60 cryopreservation in Roswell Park Memorial Institute medium (RPMI) only, RPMI + 300 µM Nig, or RPMI + 200 µM Sal. Cells were cryopreserved in RPMI/DMSO +/- Nig or Sal, and protein carbonylation was measured in duplicate (each sample is composed of three sets of HL-60 cells pooled together) 1 hour post thaw in RPMI media containing Sal or Nig. Data are expressed as mean ± standard deviation (*P value < 0.05).

As an indicator of oxidative stress, protein carbonylation assessment is widely used to reflect a major form of protein oxidation. Carbonylation assays were performed to assess the effect of CPAs on protein oxidation level post thaw. The results showed that protein carbonylation level for HL-60 cells cryopreserved in DMSO + Nig was kept at the level prior to freezing the cells and averaged 0.107±0.007 nmol/mL (Fig. 6), while Sal had no significant effect on protein oxidation level (∼0.23±0.048 nmol/mL). In the absence of cryo-additives, HL-60 cell levels of protein carbonylation/oxidation post freeze-thaw in DMSO alone were approximately 0.26±0.016 nmol/mL (Fig. 7). Finally, Nig at 300 µM showed an anti-oxidative effect by reducing non-cryopreserved HL-60 proteins carbonylation levels from 0.16 nmol/mL to 0.1 nmol/mL for cells growing in Roswell Park Memorial Institute medium (RPMI) + 300 µM Nig, while this was only reduced to 0.13 nmol/mL in the presence of 200 µM Sal (Fig. 7).

## Discussion

This is the first study aimed at establishing the proteomic and biological responses of HL-60 cells subjected to storage freezing in the presence of DMSO +/- novel CPAs. Many of the proteomic findings were validated by carrying out functional/biological assays targeting the main proteomic pathways identified. The major issue with the most commonly used permeating CPAs such as DMSO is their cytotoxicity [32], leading to low cell recovery. In the present study, HL-60 cells were incubated with Nig or Sal prior to and during cryopreservation. We subsequently identified differential proteome profiles associated with HL-60 cryopreservation in DMSO +/- CPAs. For example, the highest total number of differentially expressed proteins was found in cells cryopreserved in a combination of DMSO and Nig (37%), followed by 34% in DMSO and Sal, compared to only 29% for cells cryopreserved in DMSO alone. This suggests that these two CPAs helped to preserve cellular proteins. The bulk of previous proteome profiling studies investigating nucleated cell lines were performed either on the cells without cryopreservation [33], assessing pharmacological agent effects on specific cells [34], or by comparison of cellular proteome profiles of healthy vs diseased patients [35].

The current finding demonstrated that the HL-60 cell line cryopreserved in DMSO alone exhibited an increased level of proteins associated with oxidative stress (e.g., superoxide dismutase, acyl coA oxidase, or Hsp 70-binding protein 1), which was interesting as these were mostly reversed in the presence of Nig or Sal. Furthermore, protein deglycase, a protein known to play an important role as an oxidation sensor [36], was increased in the presence of DMSO + Sal only, suggesting the promotion of an anti-oxidative environment. These findings are in line with reports of putative stress factors related to cryopreservation [37]. Furthermore, HL-60 cells cryopreserved in DMSO only showed a higher level of lipid and protein oxidation, consistent with our proteome findings. Nevertheless, further proteomic studies on nucleated cell lines are needed to address the issues of the proteome dynamic range or the proteome profiles post cryopreservation. At this stage, the most comprehensive proteomic analysis was only performed on human nucleated cell lines prior their cryopreservation [33].

The present proteomic study showed that Nig or Sal used as CPAs for the cryopreservation of HL-60 cells can either have additive or counter-regulatory effects in comparison to DMSO. For example, in response to cryo-stress, the level of NADH-ubiquinone oxidoreductase 75 kDa subunit, known to be involved with cellular oxidative metabolism [38], was upregulated in DMSO +/- Sal and even reached higher levels in the presence of Nig. This suggests that the Nig effect is more likely to target the mitochondrial machinery [39] and reduce apoptosis, as suggested by Ricci et al [39]. We also found a differential effect of Sal and Nig (when added to DMSO) on key enzymes associated with cryo-stress. For example, LDH protein level was reduced when HL-60 cells were cryopreserved in DMSO alone, and the addition of Sal reversed its levels by increasing it up to 1.6 times.

Differential effect of CPAs on the proteomic outcome of HL-60 cell cryopreservation was also reflected in the correlation between the increases in protein levels of glutathione reductase in the presence of DMSO alone. Glutathione reductase is a critical enzyme known to promote the reductive environment by protecting cells against the damaging effects of free radicals. Surprisingly, its protein levels were not correlated with its activity, which was increased in the presence of Nig or Sal. Similar findings of poor correlation between GR or LDH activities and protein levels have been reported by Glanemann et al [40].

The heat shock 70 subunits reacted differentially to cryo-stress +/- CPAs. For example, Hsp70-binding protein 1 decreased in the presence of CPAs and increased in the presence of DMSO. In contrast, heat shock 70 kDa protein 13 was not detected when HL-60 was cryopreserved in DMSO +/- Sal. The reason for such differential expression patterns of Heat shock proteins (Hsps) is not clear but might be due to post-translational modifications (e.g., carbonylation) and differential interactions with co-chaperones that might alter their functions during cryo-stress [41].

The current findings also support the role of Sal in reducing oxidative damage by promoting oxidative DNA repair as shown for hematopoietic stem cells via the regulation of the base excision repair pathway (e.g., poly(ADP-ribose) polymerase-1) [42]. Post thaw, the level of expression of proteins associated with transcriptional activities such as Rho GTPase activating protein 27 and Ras GTPase-activating-like protein IQGAP2 were also increased by Sal in comparison to cells cryopreserved in DMSO alone. This increase in the level of proteins associated with DNA repair/replication and transcriptional activities in the presence of CPA also appeared to be mirrored by an increase in the level of proteins associated with cellular growth. The levels of epidermal growth factor receptor were decrease here by 2.1 fold in the presence of Sal, while it was undetected in the recovery phase of HL-60 cells cryopreserved in DMSO +/- Nig. This receptor is generally known to be crucial in DNA replication and cell division [43], while its levels are unchanged when cryo-preserving human ovarian tissue [43-44]. Such a regulatory element of the DNA damage signaling pathways is paramount for cell survival by controlling passage from the S to the G2/M phases of the cell cycle [44]. In line with our proteomic findings, Sal has shown a noticeable promoting effect on HL-60 cell proliferation during the recovery phase. A similar elevation in proliferative proteins was found in hepatocyte cells in response to the proliferation promoter compound perfluorooctane sulfonate [45]. On the other hand, our findings conflict with the reported effect of Sal on inducing breast cancer cell cycle arrest [46]. Such an anti-proliferative effect was previously attributed to Sal being used as an anti-hypoxia agent leading to suppression of hypoxia-induced cell proliferation [47]. Finally, in the present study we have also identified an additive effect of DMSO with Sal or Nig in enhancing some cellular functions by increasing the level of cytoskeleton proteins such as ankyrin-2, synaptotagmin-like, or microtubules (Table 1), leading to a better HL-60 cell recovery and growth post thaw.

This is the first and largest targeted study aimed at deciphering proteomic profiles associated with the cryopreservation of the nucleated human cell line (HL-60) in DMSO with and without novel cryo-additive agents such as Nig. The proteome profiles associated with HL-60 cryopreservation in DMSO +/- Nig or Sal were mostly validated at the biological level as these correlated with the corresponding biological readouts (e.g., enzymatic, oxidation, and proliferative assays). HL-60 cryopreservation in DMSO only has led to oxidative damage and subsequently validating the already known biological features associated with cryo-stress. More importantly, the addition of novel CPAs has identified a potential synergistic or differential cryoprotective effect of these CPAs in comparison to cryopreserving HL-60 cells in DMSO only. Predominantly, this study has clearly shown that Nig reduces specifically protein oxidation, while Nig or Sal both reduce lipid cryo-oxidation. The most striking finding generated by the current proteomic profiling study is that post thaw, Sal increased the level of proteins that are associated with nuclear activities and subsequently increased cell proliferation in the recovery phase. The presence of CPAs (e.g., Nig or Sal) not only enhanced HL-60 cell recovery post thaw but also significantly reduced cytotoxicity by decreasing the level of LDH activity (Fig. 6) generally used as a cytotoxicity marker [48].

In summary, identifying the relevant molecular (proteomic analysis) and functional (biological readouts) pathways affected by cryopreservation and successfully targeting the compromised pathways with novel cryoprotective agents are ways to limit cryo-damage. The present findings will contribute to enhancing cryo-media formulation and potentially lead to improving future cell- and regenerative tissue-based therapies.

## Methods

### Materials

HL-60 cells (HL-60(TB) (RRID:CVCL_A794)), RPMI-1640 media, fetal bovine serum (FBS), pencillin–streptomycin, nigerose, salidroside, sterilized filtered Dulbecco's phosphate buffer saline, trypan blue solution cell culture, DMSO, isopropanol, Tris base, urea, Hydrochloric acid (HCL), ammonium biocarbonate, acetonitrile, dithiotheritol (DTT), iodoacetamine, formic acid, radio immunoprecipitation assay (RIPA) buffer, protease inhibitor cocktail, and milli-Q water were all purchased from Sigma-Aldrich (Poole, UK). A Mr. Frosty freezing container was purchased from ThermoFisher Scientific (Waltham, MA). Certified Sep-Pak C18 cc vac cartridge was purchased from (Waters, UK). Sequence grade modified trypsin was purchased from Promega (Southampton, UK). Glutathione reductase, lactate dehydrogenase, and lipid peroxidation (MDA) assay kits were purchased from Abcam (Cambridge, UK). A protein carbonyl colorimetric assay kit was purchased from Cayman Chemical Company (Ann Arbor, MI).

### Experimental design

The study was divided into three arms (Fig. 1). Arm 1 involved culturing HL-60 cells up to 70% confluence in RPMI 1460 media, containing 10% (v/v) FBS and 50 U/mL penicillin-streptomycin. HL-60 cells were centrifuged at 100 x *g* for 5 minutes, and the medium was immediately removed. HL-60 cells were re-suspended in freezing media (10% DMSO and 90% FBS) at 10^6^ cells/mL, slowly frozen in cryogenic tubes, and stored at -80°C overnight. Next, cells were cryopreserved in either the freezing media or in liquid nitrogen. HL-60 cells were thawed in a water bath at 37°C, centrifuged at 100 x *g* for 5 minutes, and washed three times with RPMI media. Post thawing, HL-60 cells were cultured in a recovery medium containing RPMI, 20% FBS, and 5 U/mL penicillin-streptomycin; the FBS concentration was reduced to 10% 24 hours post thaw. HL-60 cells were cultured as described above for arm 1 with the exception of adding 300 µM Nig (arm 2) or 200 µM Sal (arm 3) for 24 hours prior to cryopreservation, during cryopreservation, and up to 48 hours post thaw. The selected concentrations of the cryo-additive agents (e.g., Nig or Sal) were optimized as described in Supplement S4. Cells were maintained at all times in culture at 37°C under 5% C0_2/_95% air.

For proteomic and biochemical analysis (five replicates per arm), HL-60 cells cryopreserved in DMSO +/- Nig or Sal were harvested at approximately 70% confluence prior to freezing and at 24 hours or 48 hours post thaw.

### Sample preparation for NanoLC-MS/MS analyses

Human leukemia (HL-60) cells were used as a nucleated cellular model to establish its proteome profiles when cryo-preserved in DMSO with or without novel CPAs. The experimental design was set up as described in Fig. 1. Briefly, HL-60 cells were cultured in RPMI media, cryo-preserved in freezing media (10% DMSO and 90% FBS), and recovered in RPMI media (arm 1). For arms 2 and 3, 300 µM Nig and 200 µM Sal were added respectively to the culture media 24 hours prior, during cryopreservation, and up to 48 hours post thaw. HL-60 proteins were extracted by acetone precipitation. Cell pellets were mixed with 100 µL cold (-20°C) acetone and kept at -20°C for 60 minutes to allow protein precipitation. The samples were centrifuged at 13,000 x *g* for 10 min, pellet and air-dried at room temperature for 30 minutes. Pelleted proteins were homogenized in 6 M urea buffer, vortexed, and sonicated for 2 minutes. Then, 70 mM DTT was added to samples and incubated for 30–60 minutes at room temperature. Next, 140 mM Iodoacetic acid alkylating reagent was added, followed by vortexing and incubation for 30–60 minutes at room temperature. The urea concentration was reduced by adding 775 µL milliQ water and vortexing. Protein concentrations were determined using the Bradford method. After this, 60 µg of extracted proteins were trypsinized in a 1:50 ratio, mixed carefully, and left overnight at 37°C for digestion. The next day, the reactions were stopped by adjusting the pH to <6 by adding concentrated acetic acid. The digested peptides were purified using SEP-PAK C18 purification columns.

### NanoLC-MS/MS analyses

Proteomic analyses were performed in a bi-dimensional microUPLC tandem nanoESI-HDMS^E^ platform by multiplexed data-independent acquisition experiments [27]. A 2D-RP/RP Acquity UPLC M-Class System (Waters Corporation) coupled to a Synapt G2-Si HDMS mass spectrometer (Waters Corporation) platform was used. The samples were fractionated using a one-dimension reversed-phase approach. Peptide samples (0.5 µg) were loaded into a 100 Å, 1,8μm, 75 μm × 150 mm M-Class HSS T3 column (Waters Corporation). The fractionation was achieved by using an acetonitrile gradient from 7% to 40% (v/v) over 95 minutes at a flow rate of 0.4 µL/min directly into a Synapt G2-Si mass spectrometer. For every measurement, the mass spectrometer was operated in resolution mode with an m/z resolving power of about 20,000 FWHM, using ion mobility with a cross-section resolving power of at least 40 Ω/ΔΩ. MS and MS/MS data were acquired in positive ion mode using ion mobility separation of precursor ions (HDMS^E^) over a range of 50–2000 m/z. The lock mass channel was sampled every 30 seconds. The mass spectrometer was calibrated with a MS/MS spectrum of [Glu1]-fibrinopeptide B human solution delivered through the reference sprayer of the NanoLock Spray source.

### Data processing and database searches

Proteins were identified and quantified by using dedicated algorithms and searching against the Uniprot proteomic database of *Homo sapiens* (version 2016/09) [49]. The databases used were reversed “on the fly” during its queries and appended to the original database to assess the false-positive identification rate. For proper spectral processing, database searching and label free quantification, we used Progenesis QI for Proteomics software package with Apex3D, Peptide 3D, and Ion Accounting informatics (Waters Corporation). This software starts with loading of the LC-MS data, followed by alignment and peak detection, which creates a list of interesting peptide ions that are explored within Peptide Ion Stats by multivariate statistical methods. The processing parameters used were 150 counts for the low-energy threshold, 50.0 counts for the elevated energy threshold, and 750 counts for the intensity threshold. Automatic alignment of the runs (all runs in the experiment were assessed for suitability) was used for the processing. In peak picking, 8 was used as maximum ion charge, and the sensitivity value was set as 4. Moreover, the following parameters were considered in identifying peptides: digestion by trypsin with at most two missed cleavages; variable modifications by oxidation (M) and fixed modification by carbamidomethyl (C); false discovery rate less than 1%. One or more ion fragments per peptide, three or more fragments per protein, and one or more peptides per protein were required for ion matching. Identifications that did not satisfy these criteria were rejected. The experimental design is summarized in Fig. 1 (see arm 1, arm 2, and arm 3), and the label free protein quantitation was done using the Hi-N (N = 3) method [49]. The Shapiro–Wilk W-test analysis of variance was used to identify proteins that were present at different levels. Only those findings with *P* values < 0.05 were considered as significant. Finally, proteins with mean changes of 1.5 fold were considered as differentially expressed.

## Validation Assays

### Enzymatic activities

HL-60 cell pellets were collected and washed in cold phosphate-buffered saline once as described above and lysed in 350 µL RIPA buffer and 2.85 µL protease inhibitors and kept on ice for 30 minutes. Cell lysates were centrifuged at 100 x *g* for 5 minutes, and enzymatic assays were performed using an amount equivalent to 1 × 10^6^ HL-60 cells according to the manufacturer's instructions. The GR assay is based on measuring spectrophotometrically the resulting chromophore (TNB) (e.g., sulfhydryl-glutathione and 5,5’-dithiobis [2-nitrobenzoic acid]) at 405 nm. The first and second readouts were measured at 5- and 10-minute intervals using the Spectrostar Nano plate reader (Promega). LDH assays were also performed according to the manufacturer's instructions. The quantity of NADH was detected spectrophotometrically at 450 nm by mixing NADH detection buffer with the cell supernatant and lysate. The first readout was taken immediately, and the samples were incubated in the dark at 37°C with a final colorimetric reading at 30 minutes.

### Protein and lipid oxidation assays

Protein oxidation or carbonylation was measured in two sets of samples (each sample is composed of 3 sets of HL-60 cells pooled together) prior to cryopreservation and 24 hours post thaw. The carbonylation assay was performed according to the manufacturer's instructions. Briefly, a reaction between 2,4-dinitrophenylhydrazine and oxidized carbonyl groups on proteins was conducted using Cayman's protein assay kit. The derivatized carbonyl groups were quantitated by reading spectrophotometrically at 375 nm. For lipid peroxidation, measurements were carried out in triplicate on amounts equivalent to 10^6^ cells/mL by identifying the formation of MDA-thiobarbituric acid adduct in acidic conditions at 95°C for 1 hour. Sample absorbances were measured at 532 nm using the Spectrostar nano plate reader following the manufacturer's instructions. The MDA concentration was expressed in nmol.

### Cell proliferation

HL-60 cell viability and proliferation were assessed at 1 hour, 24 hours, and 48 hours post thaw. Cells were mixed with trypan blue and placed on hemocytometer slides for counting under light microscope in duplicate at each time point.

### Statistical analyses

All enzymatic assays were performed using five biological replicates. The lipid oxidation assay was performed in triplicate, and the protein carbonylation assay was carried out in duplicate. Results were presented as mean ± standard deviation. Significant differences between groups were determined using Student *t*test for paired and unpaired observations. *P* values <0.05 were considered significant.

## Availability of supporting data

The mass spectrometry proteomics data have been deposited to the ProteomeXchange Consortium via the PRIDE partner repository with the dataset identifier PXD006998. Additional supporting data are available in the *GigaScience* GigaDB repository [50].

## Additional files

**Table S1:** Label-free LCMS/MS proteome analysis of Human promyelocytic leukemia HL-60 cells cryopreserved in DMSO [n = 5 replicates].

**Table S2:** Label-free LCMS/MS proteome analysis of Human promyelocytic leukemia HL-60 cells cryopreserved in DMSO + Nig [n = 5 replicates].

**Table S3:** Label-free LCMS/MS proteome analysis of Human promyelocytic leukemia HL-60 cells cryopreserved in DMSO + Sal [n = 5 replicates].

**Figure S4:** CPAs dose response. The effect of Nig and Sal at different concentrations on HL-60 cell viability post cryopreservation in 10% DMSO +/- Nig or Sal. HL-60 cell cryosurvival was measured in triplicate using trypan blue.

## Abbreviations

CPA: cryo-protective agent; DMSO: dimethylsulfoxide; DTT: dithiotheritol; FBS: fetal bovine serum; GR: glutathione reductase; HL-60: human leukemia cells; LCMS/MS: liquid chromatography-high resolution mass spectrometry/mass spectrometry; LDH: lactate dehydrogenase; MDA: malondialdehyde; Nig: nigerose; RIPA: radio immunoprecipitation assay; RPMI: Roswell Park Memorial Institute; Sal: salidroside.

## Competing interests

The authors declare that they have no competing interests.

## Funding

This work was supported by the King Abdul Aziz City for Science and Technology research fund. J.S.C. and D.,M.S. are funded by FAPESP (São Paulo Research Foundation, grants 2014/14 881–1, 2013/0 8711–3, and 2014/10 068–4) and CNPq (the Brazilian National Council for Scientific and Technological Development, grant 460 289/2014–4).

## Author contributions

N.A.S.A. performed all experimental manipulations and sample preparation for mass spectrometry, prepared the tables and figures, and performed bioinformatic analysis. J.S.C. performed sample acquisition and data analysis mass spectrometry. D.M. supervised the proteomics pipeline. N.K.H.S. co-supervised the project. H.R. designed and supervised the project and performed biological interpretation of the data. N.A.S.A., J.S.C., D.M., N.K.H.S., and H.R. wrote the manuscript. All authors edited and approved the final version of the manuscript.

## Supplementary Material

GIGA-D-18-00064_Original_Submission.pdf

GIGA-D-18-00064_Revision_1.pdf

GIGA-D-18-00064_Revision_2.pdf

GIGA-D-18-00064_Revision_3.pdf

Response_to_Reviewer_Comments_Original_Submission.pdf

Response_to_Reviewer_Comments_Revision_1.pdf

Response_to_Reviewer_Comments_Revision_2.pdf

Reviewer_1_Report_(Original_Submission) -- Stefania Angelucci 4/8/2018 Reviewed

Reviewer_1_Report_Revision_1 -- Stefania Angelucci 9/3/2018 Reviewed

Reviewer_2_Report_(Original_Submission) -- Rene Zahedi 4/27/2018 Reviewed
