# Supplementary material for: Human leukemia cells (HL-60) proteomic and biological signatures underpinning cryo-damage are differentially modulated by novel cryo-additives
Source: Gigascience. 2018 Dec 10;8(3):giy155. doi: 10.1093/gigascience/giy155 (PMC6394207; doi:10.1093/gigascience/giy155)
Supplement: GIGA-D-18-00064_Original_Submission.pdf [file giy155_giga-d-18-00064_original_submission.pdf]

GigaScience

Human Leukaemia cells (HL-60) proteomic and biological signatures underpinning cryo-damage are differentially modulated by novel cryo-additives.

--Manuscript Draft--

**Manuscript Number:**	GIGA-D-18-00064
**Full Title:**	Human Leukaemia cells (HL-60) proteomic and biological signatures underpinning cryo-damage are differentially modulated by novel cryo-additives.
**Article Type:**	Research
**Funding Information:**	King Abdulaziz City for Science and Technology — Dr Nigel Slater<br>Fundação de Amparo à Pesquisa do Estado de São Paulo (2014/14881-1) — Dr Daniel Martins-de-Souza<br>Brazilian National Council for Scientific and Technological Development (460289/2014-4) — Dr Daniel Martins-de-Souza
**Abstract:**	Background: Cryopreservation is a routinely used methodology for prolonged storage of viable cells. The use of cryo-protective agents (CPAs) such as dimethylsulfoxide (DMSO), glycerol or trehalose is paramount to reduce cellular cryo-injury but their effectiveness is still limited. The current study focuses on establishing and modulating the proteomic and the corresponding biological profiles associated with the cryo-injury of human leukaemia (HL-60) cells cryopreserved in DMSO alone or DMSO +/- novel CPAs [e.g. nigerose (Nig) or salidroside (Sal)].<br><br>Findings: To reduce cryo-damage, HL-60 cells were cultured prior and post cryopreservation in RPMI-1640 media +/- Nig or Sal. Shotgun proteomic analysis showed significant alterations in the levels of proteins in cells cryopreserved in Nig or Sal compared to DMSO. Nig mostly affected cellular metabolism and energy pathways, whereas Sal increased the levels of proteins associated with DNA repair/duplication, RNA transcription and cell proliferation. Validation testing showed that the proteome profile associated with Sal was correlated with a 2.8 fold increase in cell proliferative rate. At the functional level, both Nig and Sal increased glutathione reductase (0.0012±6.19E-05 and 0.0016±3.04E-05 mU/mL, respectively) compared to DMSO controls (0.0003±3.7E-05 mU/mL) and reduced lactate dehydrogenase activities (from -2.5 to -4.75 fold) and lipid oxidation (-1.6 fold). In contrast, only Nig attenuated protein carbonylation.<br><br>Conclusions: We have identified key molecules and corresponding functional pathways underpinning the effect of cryopreservation (+/- CPAs) of HL-60 cells. We also validated the proteomic findings by identifying the corresponding biological profiles associated with promoting an anti-oxidative environment post cryopreservation. Nig or Sal in comparison to DMSO showed a differential or additive effects in regards to reducing cryo-injury and enhancing cell survival/proliferation post thaw. These results can provide useful insight to cryo-damage and the design of enhanced cryomedia formulation.
**Corresponding Author:**	Hassan Rahmoune, Ph. D.<br>University of Cambridge<br>UNITED KINGDOM
**Corresponding Author Secondary Information:**	
**Corresponding Author's Institution:**	University of Cambridge
**Corresponding Author's Secondary Institution:**	
**First Author:**	Hassan Rahmoune, Ph. D.
**First Author Secondary Information:**	

Powered by Editorial Manager® and ProduXion Manager® from Aries Systems Corporation

**Order of Authors:**	Hassan Rahmoune, Ph. D.
	Noha Al-Otaibi
	Juliana Cassoli
	Daniel Martins-de-Souza
	Nigel Slater
**Order of Authors Secondary Information:**	
**Opposed Reviewers:**	
**Additional Information:**	
**Question**	**Response**
Are you submitting this manuscript to a special series or article collection?	No
**Experimental design and statistics**<br><br>Full details of the experimental design and statistical methods used should be given in the Methods section, as detailed in our Minimum Standards Reporting Checklist. Information essential to interpreting the data presented should be made available in the figure legends.<br><br>Have you included all the information requested in your manuscript?	Yes
**Resources**<br><br>A description of all resources used, including antibodies, cell lines, animals and software tools, with enough information to allow them to be uniquely identified, should be included in the Methods section. Authors are strongly encouraged to cite Research Resource Identifiers (RRIDs) for antibodies, model organisms and tools, where possible.<br><br>Have you included the information requested as detailed in our Minimum Standards Reporting Checklist?	Yes
**Availability of data and materials**<br><br>All datasets and code on which the conclusions of the paper rely must be either included in your submission or deposited in publicly available repositories (where available and ethically appropriate), referencing such data using a unique identifier in the references and in the "Availability of Data and Materials"	Yes

section of your manuscript.<br><br>Have you have met the above requirement as detailed in our Minimum Standards Reporting Checklist?	

Manuscript

Click here to download Manuscript Noha et al HL-60 GigaScience 2018.pdf

Human Leukaemia cells (HL-60) proteomic and biological signatures underpinning cryo-damage are differentially modulated by novel cryo-additives

Noha A. S. Al-Otaibi[1,2], Juliana S. Cassoli[3], Daniel Martins-de-Souza[3], Nigel K. H. Slater[1], Hassan Rahmoune[1 #]

(1) Department of Chemical Engineering & Biotechnology, University of Cambridge, Philippa Fawcett Drive. Cambridge CB3 0AS, United Kingdom.

(2) King Abdulaziz City for Science and Technology Kingdom of Saudi Arabia P.O Box 6086, Riyadh 11442, Saudi Arabia.

(3) Laboratory of Neuroproteomics, Department of Biochemistry and Tissue Biology Institute of Biology, University of Campinas (UNICAMP), Campinas, SP, Brazil.

[#] Address correspondence to Hassan Rahmoune, Ph. D., (1). Email: hr228@cam.ac.uk

E-mail addresses (in the order of appearance): naa37@cam.ac.uk, jscassoli@gmail.com, dmsouza@unicamp.br, nkhs2@cam.ac.uk, hr228@cam.ac.uk

Running Title: Modulating molecular profiles underpinning HL-60 cryo-damage

Abstract

Background: Cryopreservation is a routinely used methodology for prolonged storage of viable cells. The use of cryo-protective agents (CPAs) such as dimethylsulfoxide (DMSO), glycerol or trehalose is paramount to reduce cellular cryo-injury but their effectiveness is still limited. The current study focuses on establishing and modulating the proteomic and the corresponding biological profiles associated with the cryo-injury of human leukaemia (HL-60) cells cryopreserved in DMSO alone or DMSO +/- novel CPAs [e.g. nigerose (Nig) or salidroside (Sal)].

Findings: To reduce cryo-damage, HL-60 cells were cultured prior and post cryopreservation in RPMI-1640 media +/- Nig or Sal. Shotgun proteomic analysis showed significant alterations in the levels of proteins in cells cryopreserved in Nig or Sal compared to DMSO. Nig mostly affected cellular metabolism and energy pathways, whereas Sal increased the levels of proteins associated with DNA repair/duplication, RNA transcription and cell proliferation. Validation testing showed that the proteome profile associated with Sal was correlated with a 2.8 fold increase in cell proliferative rate. At the functional level, both Nig and Sal increased glutathione reductase (0.0012±6.19E-05 and 0.0016±3.04E-05 mU/mL, respectively) compared to DMSO controls (0.0003±3.7E-05 mU/mL) and reduced lactate dehydrogenase activities (from -2.5 to -4.75 fold) and lipid oxidation (-1.6 fold). In contrast, only Nig attenuated protein carbonylation.

Conclusions: We have identified key molecules and corresponding functional pathways underpinning the effect of cryopreservation (+/- CPAs) of HL-60 cells. We also validated the proteomic findings by identifying the corresponding biological profiles associated with promoting an anti-oxidative environment post cryopreservation. Nig or Sal in comparison to DMSO showed a differential or additive effects in regards to reducing cryo-injury and enhancing cell survival/proliferation post thaw. These results can provide useful insight to cryo-damage and the design of enhanced cryomedia formulation.

Keywords: Cryopreservation, Oxidative stress, Dimethylsulfoxide, Nigerose, Salidroside.

Background

Cryopreservation of viable cells and tissues is a powerful approach to ensure cell longevity and integrity and facilitate cell/tissue engineering therapy [1]. Cell-based therapy is a rapidly emerging industry and is estimated to be worth around $5 billion in the USA alone [2]. Despite well-established cryopreservation protocols, cells remain subject to a high level of cryo-damage leading to compromised cell function and necrosis [3]. The cellular damage is generally seen as lipid and protein oxidation, which can severely affect cell stability [4] and ability to proliferate [5]. Thus, reducing the impact of cryo-damage is paramount to enhance cell recovery rate post freeze/thaw cycles.

Despite their reported toxic properties, DMSO and glycerol are the most commonly used cryo-protective agents (CPAs) to reduce cryo-injury and increase cell viability [5]. Other CPAs such as trehalose have been used for their cryo-protective properties against intracellular ice crystal formation [6]. However, the protective effect of these compounds is still limited [7] with low cell viability and recovery rates post cryopreservation [8]. The use of CPAs can also lead to production of reactive oxygen species, whereby cells are subjected to oxidative damage during freeze-thaw cycles [9]. Moreover, the effectiveness of intracellular or auto anti-oxidative response to cryo-insult is limited as cell survival is reduced [10]. Attempts to promote cellular anti-oxidative status have been reported before and these showed an improved cell survival rate [11]. For example, the use of arabidopsis thaliana containing high levels of ascorbic acid increased intracellular catalase activity leading to a higher cell survival rate post thaw [11].

The majority of studies on cryopreservation have focused on either fertility [12-14] or stem cells [5] although limited attempts have been carried out to improve cryopreservation of cell lines (e. g. Hepatocytes) [15]. Moreover, the bulk of empirical studies attempting to decipher molecular profiles associated with cryo-injury have been conducted mainly on fertility-related specimens [16, 17], plant cells [18] or stem cells [19]. Likewise, attempts to modify cryo-proteomic profiles using CPAs or DMSO +/- antifreeze have been made mainly in the field of reproductive medicine [20, 21]. In

contrast, only a limited number of molecular/functional studies have been conducted on nucleated-human cell lines to decipher and modulate biological pathways underpinning cryo-damage.

Here, we have used human leukemia (HL-60) cells as a nucleated cellular model to establish the biomolecular profiles associated with cryo-damage in the presence of DMSO alone or with the addition of salidroside (Sal) or the novel CPA nigerose (Nig) [4]. The addition of Sal with the tyrosol glucoside, as the active component of the herb Rhodiolarosea, was used previously to prevent high altitude sickness [22]. Sal has also been found to act as antioxidant against hydrogen peroxide-induced apoptosis of human red blood cells [23] and as a CPA for red blood cell cryopreservation [4]. However, this is the first investigation to test the potential cryoprotective properties of Nig. Nig is an un-fermentable sugar obtained by partial hydrolysis of nigeran and is polyol extracted from fermentation of microorganisms such as black mold or dextrans [24] as well as honey [25]. A hypothesis driven approach is clearly needed here to elucidate and modify cell-specific molecular and biological pathways associated with cyo-injury. Here we have employed a shotgun proteomics approach to profile and modulate the molecular pathways underpinning human nucleated cell cryo-damage. The present study also offers the opportunity to enhance future cryomedia formulation, minimize losses of cell viability and maximize cell recovery post freeze-thaw cycle.

Data Description

Proteins were identified and quantified by using dedicated algorithms and searching against the Uniprot proteomic database of *Homo sapiens* (version 2016/09), with the default parameters for ion accounting [26]. The databases used were reversed “on the fly” during the database queries and appended to the original database to assess the false-positive identification rate. For proper spectral processing and database searching conditions, we used Progenesis QI for Proteomics software package with Apex3D, Peptide 3D, and Ion Accounting informatics (Waters Corporation). The label free protein quantitation was done using Hi-N (N=3) method [27]. This software starts with LC-MS data loading and then performs alignment and peak detection, which creates a list of interesting peptide ions (peptides) that are explored within Peptide Ion Stats by multivariate statistical methods. The initial ion-matching

requirements were ≧1 fragment per peptide, ≧3 fragments per protein and ≧1 peptide per protein. The following parameters were considered in identifying peptides: 1) digestion by trypsin with at most two missed cleavages; 2) variable modifications by oxidation (M) and glycosylation (-O-GlcNac ST) and fixed modification by carbamidomethyl (C); and 3) false discovery rate (FDR) less than 1%. Identifications that did not satisfy these criteria were rejected. The Shapiro–Wilk W-test analysis of variance (ANOVA) was used to identify proteins that were present at different levels. Only those findings with *p*-values <0.05 were considered as significant.

Analyses

Proteins found to present at significantly different levels in HL-60 cells cryopreserved in DMSO alone, DMSO+Nig or DMSO+Sal (n=5) were classified according to their biological and functional pathways. The Uniprot accession codes of differentially expressed proteins or genes were mapped to Gene Ontology Annotation using a software linked to Funrich database. (http:// www.funrich.org) [28]. Unique and overlapping differentially expressed (e. g. up/down-regulated) proteins of HL-60 cells cryopreserved in DMSO +/- Nig or Sal are illustrated in **Figure 2**.

Proteomic Analyses

Label-free quantitative shotgun proteomic analysis was used to identify HL-60 cell proteins found at different levels in a comparison of post cryopreservation in DMSO alone, DMSO +Nig or DMSO + Sal. The effect of cryopreservation showed a higher number of significantly quantified proteins for DMSO +Nig (1,140 proteins – **Figure 2A**) and DMSO + Sal (1,032 proteins - Supplementary Tables **Figure 2A**), with only 887 found for DMSO alone (**Figure 2A**). In addition, the number of uniquely identified proteins was 158, 224 and 231 in DMSO, DMSO + Nig and DMSO + Sal, respectively. *In silico* functional analysis of the proteomes revealed the nature of biological pathways associated with cryo-damage of HL-60 cryopreserved in DMSO alone and those which were differentially modulated by the CPAs post thaw. A proportionately high number of proteins (21.05%) engaged in nucleotide and nucleobase regulation or DNA binding were identified in HL-60 cells cryopreserved in DMSO + Sal.

In contrast, the DMSO + Nig arm showed the highest proportion of changes (16.8%) in proteins associated with energy pathways and protein metabolism (**Figure 3A**). Supplementing DMSO with Nig or Sal as CPAs also led to an increased level of proteins with oxidoreductase activities, especially in the case of Nig (**Figure 3B**). The level of proteins linked to cell maintenance was the highest in HL-60 cells cryopreserved in DMSO alone (12.5%) when compared to DMSO +Nig (8%) and DMSO + Sal (6.4%).

The percentage of recognised DNA binding proteins were estimated at 8.09% for cells cryopreserved in DMSO + Sal while this did not exceed 2% in cells cryopreserved in DMSO +Nig and DMSO alone (**Figure 3B**). HL-60 protease activity-associated proteins were estimated at 4.4% in DMSO +Nig, 3.1% in DMSO alone, while only reaching 2.02% in DMSO + Sal (**Figure 3B**). With regards to cryo-stress, heat shock proteins were differentially expressed in HL-60 cells cryopreserved in DMSO + Sal (1.2%) and DMSO alone (0.6%), whereas these proteins were not detected in cells cryopreserved in the presence of Nig. The proteome profile reflecting the effect of freeze/thaw cycle on HL-60 cells cryopreserved in DMSO alone and DMSO + Nig or Sal are summarised in **Table 1**.

Oxido-redox functions (Table 1)

Reduction in HL-60 cryo-oxidation was shown by an increased level of glutathione reductase and superoxide dismutase [Cu-Zn] by 3.2 ad 1.4 fold, respectively, in DMSO alone (**Table 1**). However, no significant change was detected in the levels of either of these markers for cells preserved in the presence of Nig or Sal. In contrast, the levels of thioredoxin reductase-1 were increased up to 35 fold when Nig was added and by 15 fold with the addition of Sal. A similar pattern was seen with the NADH-ubiquinone oxidoreductase 75 kDa subunit. The level of pro-oxidative enzymes were all reduced in the presence of CPAs such as peroxiredoxin (not detected in DMSO, downregulated by 2.0-fold in DMSO + Nig and by 3.5-fold in DMSO + Sal), glutathione S-transferase Kappa-1 (decreased by 8-fold in DMSO, decreased 13.6-fold in DMSO + Nig and decreased 3.5-fold in DMSO +Sal) and thioredoxin-dependent peroxide reductase [decreased3.0-fold in DMSO, decreased 5.2 fold in DMSO + Nig and

decreased 8.8-fold in DMSO + Sal). Very long-chain specific acyl-CoA dehydrogenase (involved in fatty acid β-oxidation) showed a 4-fold decreased level in HL-60 cells cryopreserved in DMSO + Nig and a 5-fold decrease in DMSO + Sal compared to the levels in cells cryopreserved in DMSO alone. A similar anti-oxidative pattern was observed in the presence of CPAs with increased levels of acyl-coenzyme A oxidase (16.8 fold in DMSO + Nig and 42.7-fold in DMSO + Sal) and carbonyl oxidase (5.5 fold in the presence of DMSO + Sal).

A differential response to cryo-stress was identified when Nig or Sal were added to media prior to and post cryopreservation of HL-60 cells. For example, the stress-related protein Hsp 70-binding protein 1 was increased 14.4-fold in HL-60 cells cryopreserved in DMSO alone but its level decreased by 71- and 77-folds in the presence of Nig or Sal respectively. In contrast, cytosolic stress response proteins such as the heat shock 70 kDa protein 4 was not detected in DMSO +/- Nig and was increased by 2.3-fold in DMSO + Sal. Finally, microsomal Hsp 70 protein-13 was not detected in HL-60 cells cryopreserved in DMSO +/- Sal while this same protein was increased 15.8-fold in the presence of DMSO + Nig.

Nuclear and cellular functions *(Table 1)*

Twenty-four hours post thaw, incubation of HL-60 cell in Sal led to a marked increase in its nuclear proteins as shown in **Table 1**. In the presence of Sal, the levels of proteins associated with DNA repair were increased such as DNA excision repair protein ERCC-6-like (8.8-fold in Sal, not detected in the presence of DMSO + Nig and decreased by 14.4-fold in DMSO alone), mini-chromosome maintenance complex-binding protein (increased by 71-fold in DMSO + Sal, 11-fold in DMSO +Nig and not detected in DMSO alone). Sal also enhanced the levels of proteins involved in transcriptional regulation such as transcription factor TFIIIB component B protein (increased by 11-fold in DMSO + Sal, 8-fold in DMSO + Nig, and by 2-fold in DMSO alone).

In the presence of CPAs, the significantly altered levels of proteins associated with nuclear activities were reflected by the changes in proteins associated with cell growth and cytosolic functions. For example, the presence of Sal and Nig doubled the fold change of cyclin-G-associated kinase from a 4-fold increase in DMSO alone, up to 8 or 9-fold increase in Nig and Sal, respectively. TBC1 domain

family member 2A, known to be involved in the regulation of GTPase activities and vesicle fusion, was not detected post thaw for HL-60 cryopreserved in DMSO alone while it was increased in the presence of Nig by 11.2-fold and up to 39-fold in Sal. The levels of cytoskeletal proteins were also increased by the CPAs such as ankyrin-2, microtubules-associated protein and echinoderm microtubule-associated protein-like 1) which are known to be associated with cell shape. Functions such as cell re-organisation and division were also increased in the presence of Nig and Sal compared to DMSO alone (Table1).

HL-60 cell proliferation post thaw *(Table 1)*

The number of HL-60 cells 24 h post thaw was estimated at 265 x 10^4, 130 x 10^4 and 180 x 10^4 cells/mL for DMSO alone, DMSO + Nig and DMSO + Sal respectively (**Figure 4**). At 48 h, Sal increased the proliferative rate by 2.84-fold compared to cells cryopreserved in DMSO alone and this was 1.3-fold for DMSO + Nig compared to cells cryopreserved in DMSO alone (640 x 10^4 cells/mL). The direct comparison between the effect of Nig and Sal on cell growth rate at 48 h showed that the number of HL-60 cells in the presence Sal was at 1820 x 10^4 cells/mL while this only reached 860 x10^4 cells/mL in the presence of Nig. Such an increase in the HL-60 cell proliferative rate post thaw in the presence of Sal was paralleled by the increase in the protein levels of epidermal growth factor receptor (2.1-fold) and cyclin-G-associated kinase (9.8-fold) (**Table 1**).

Biological profiles of HL-60 cryopreserved in DMSO +/- Nig or Sal

HL-60 cell intracellular glutathione reductase (GR) activity was measured [n=5] prior to freezing and 24 h post thaw. GR activity was significantly increased in all cases. The presence of CPAs in the media significantly boosted GR activity from 0.0003 mU/mL prior to cryopreservation to 0.0005 mU/mL in the presence of DMSO alone. The addition of Nig boosted GR activity post thaw even further reaching 0.0013±0.00006 mU/mL. Sal had the biggest effect on HL-60 cell GR activity with a reading of 0.0016 mU/mL (i.e. 3 times more increased compared to HL-60 cells cryopreserved in the standard DMSO cryomedia). HL-60 cell intracellular Lactate dehydrogenase (LDH) activities were also measured prior to freezing and 24 h post thaw [n=5]. Adding Sal to the culture or cryomedia lowered LDH readouts

from 0.1±0.03 mU/mL in DMSO alone to 0.04±0.01 mU/mL in DMSO + Sal. Moreover, the addition of Nig had the biggest effect on lowering LDH activity by bringing this to 0.02±0.044 mU/mL (3 times lower than prior to cryopreservation, 5 times less than DMSO alone and 2 time less than DMSO + Sal).

HL-60 lipid peroxidation level was measured in triplicate prior to freezing, and 1 h and 24 h post thaw in the presence and absence of Nig or Sal. Measurement of MDA levels 1 h post thaw showed a significant increase in lipid oxidation with HL-60 cells cryopreserved in DMSO alone reaching an level of 7.31±0.16 nmol/mL (**Figure 5**). In contrast, this was approximately 40% lower in the presence of Nig (4.35±0.02 nmol/mL) or Sal (4.53±0.09 nmol/mL). In the recovery phase (e.g. 24 h post thaw), HL-60 cell lipid peroxidation levels reached control levels (e.g. prior to cryopreservation ~ 2.1 nmol/mL). One day post thaw, lipid oxidation levels for HL-60cells cryopreserved in DMSO +/- Nig or Sal reversed back to its prior cryopreservation level (**Figure 5**).

The results showed that protein carbonylation level for HL-60 cells cryopreserved in DMSO + Nig was kept at the level prior to freezing the cells and averaged 0.107±0.007 nmol/mL (**Figure 6**) while Sal had no significant effect (~ 0.23±0.048 nmol/mL). In the absence of cryo-additives, HL-60 cell levels of protein carbonylation post freeze-thaw in DMSO alone were approximately 0.26±0.016 nmol/mL (**Figure 7**). Finally, Nig at 300 µM showed an anti-oxidative effect by reducing non-cryopreserved HL-60 proteins carbonylation levels from 0.16 nmol/mL to 0.1 nmol/mL for cells growing in RPMI + 300 µM Nig, while this was only reduced to 0.13 nmol/mL in the presence of 200 µM Sal (**Figure 7**).

Discussion

This is the first study aimed at establishing the proteomic and biological responses of HL-60 cells subjected to storage freezing in the presence of DMSO +/- novel CPAs. Many of the proteomic findings were validated by carrying out functional/biological assays targeting the main proteomic pathways identified. The major issue with the most commonly used permeating CPAs such as DMSO is their

cytotoxicity [29], leading to low cell recovery. In the present study, HL-60 cells were incubated with Nig or Sal prior to and during cryopreservation. We subsequently identified differential proteome profiles associated with HL-60 cryopreservation in DMSO +/- CPAs. For example the highest total number of differentially expressed proteins was found in cells cryopreserved in a combination of DMSO and Nig (37%), followed by 34% in DMSO and Sal, compared to only 29% for cells cryopreserved in DMSO alone. This suggests that these two CPAs helped to preserve cellular proteins. The bulk of previous proteome profiling studies investigating nucleated cell lines were either performed on the cells without cryopreservation [30], assessing pharmacological agent effects on specific cells [31] or comparison of cellular proteome profiles of healthy versus diseased patients [32].

The current finding demonstrated that the HL-60 cell line cryopreserved in DMSO alone exhibited an increased level of proteins associated with oxidative stress (e. g. superoxide dismutase, acyl coA oxidase or Hsp 70-binding protein 1) was interesting as these were mostly reversed in the presence of Nig or Sal. Furthermore, protein deglycase, a protein known to play an important role as an oxidation sensor [33], was increased in the presence of DMSO + Sal only, suggesting the promotion of an anti-oxidative environment. These findings are in line with reports of putative stress factors related to cryopreservation [34]. Furthermore, HL-60 cells cryopreserved in DMSO only showed a higher level of lipid and protein oxidation, consistent with our proteome findings.

The present proteomic study showed that Nig or Sal used as CPAs for the cryopreservation of HL-60 cells can either have additive or counter-regulatory effects in comparison to DMSO. For example, in response to cryo-stress, the level of NADH-ubiquinone oxidoreductase 75 kDa subunit, known to be involved with cellular oxidative metabolism [35], was upregulated in DMSO +/- Sal and even reached higher levels in the presence of Nig. This suggests that the Nig effect is more likely to target the mitochondrial machinery and reduce apoptosis as suggested by Ricci et al [36]. We also found a differential effect of Sal and Nig (when added to DMSO) on key enzymes associated with cryo-stress. For example, of LDH protein level was reduced when HL-60 cells were cryopreserved in DMSO alone and the addition of Sal reversed its levels by increasing it up to 1.6 times.

Differential effect of CPAs on the proteomic outcome of HL-60 cell cryopreservation was also reflected in the correlation between the increases in protein levels of glutathione reductase in the presence of DMSO alone. Glutathione reductase is a critical enzyme known to promote the reductive environment by protecting cells against the damaging effects of free radicals. Surprisingly, its protein levels were not correlated with its activity, which was increased in the presence of Nig or Sal. Similar findings of poor correlation between GR or LDH activities and protein levels have been reported elsewhere by Glanemann et al [37].

The heat shock 70 subunits reacted differentially to cryo-stress +/- CPAs. For example, Hsp70-binding protein 1 decreased in the presence of CPAs and increased in the presence of DMSO. In contrast, Heat shock 70 kDa protein 13 was not detected when HL-60 was cryopreserved in DMSO +/- Sal. The reason for such differential expression patterns of Hsps is not clear but might be due to post-translational modifications (e.g. carbonylation) and differential interactions with co-chaperones which might alter their functions during cryo-stress [38].

The current findings also support the role of Sal in reducing oxidative damage by promoting oxidative DNA repair as shown for hematopoietic stem cells via the regulation of the base excision repair pathway (e.g. poly(ADP-ribose) polymerase-1) [39]. Post thaw, the level of expression of proteins associated with transcriptional activities such as Rho GTPase activating protein 27 and Ras GTPase-activating-like protein IQGAP2 were also increased by Sal in comparison to cells cryopreserved in DMSO alone. This increase in the level of proteins associated with DNA repair/replication and transcriptional activities in the presence of CPA also appeared to be mirrored by an increase in the level of proteins associated with cellular growth. For example, the levels of epidermal growth factor receptor were increased by 2.1-fold in the presence of Sal, while it was undetected in the recovery phase of HL-60 cells cryopreserved in DMSO +/- Nig. This receptor is known to be crucial in DNA replication and cell division [40]. Such a regulatory element of the DNA damage signalling pathways is paramount for cell survival by controlling passage from the S to the G2/M phases of the cell cycle [41]. In line with our proteomic findings, Sal has shown a noticeable promoting effect on HL-60 cell proliferation during the

recovery phase. A similar elevation in proliferative proteins was found in hepatocyte cells in response to the proliferation promoter compound perfluorooctane sulfonate [42]. On the other hand, our findings conflict with the reported effect of Sal on inducing breast cancer cell cycle arrest [43]. Such an anti-proliferative effect was previously attributed to Sal being used as anti-hypoxia agent leading to suppression of hypoxia-induced cell proliferation [44]. Finally, in the present study we have also identified an additive effect of DMSO with Sal or Nig in enhancing some cellular functions by increasing the level of cytoskeleton proteins such as ankyrin-2, synaptotagmin-like or microtubules (**Table 1**) leading to a better HL-60 cell recovery and growth post thaw.

This is the first and largest targeted study aimed at deciphering proteomic profiles associated with the cryopreservation of the nucleated human cell line (HL-60) in DMSO with and without novel cryo-additives agent such as Nig. The proteome profiles associated with HL-60 cryopreservation in DMSO +/- Nig or Sal were mostly validated at the biological level as these correlated with the corresponding biological readouts (e.g. enzymatic, oxidation and proliferative assays). HL-60 cryopreservation in DMSO only has led to oxidative damage and subsequently validating the already known biological features associated with cryo-stress. More importantly, the addition of novel CPAs has identified a potential synergistic or differential cryoprotective effect of these CPAs in comparison to cryopreserving HL-60cells in DMSO only. Predominantly, this study has clearly shown that Nig reduces specifically protein oxidation while Nig or Sal both reduce lipid cryo-oxidation. The presence The most striking finding generated by the current proteomic profiling study is that post thaw, Sal increased the level of proteins that are associated with nuclear activities and subsequently increased cell proliferation in the recovery phase.

In summary, identifying the relevant molecular (Proteomic analysis) and functional (biological readouts) pathways affected by cryopreservation and successfully targeting the compromised pathways with novel cryoprotetive agents is a way forward to limit cryo-damage. The present findings will contribute to enhancing cryo-media formulation and potentially lead to improving future cell based therapies.

Methods

Materials

HL-60 cells, RPMI-1640 media, fetal Bovine bovine serum (FBS), pencillin –streptomycin, nigerose, salidroside, sterilised filtered dulbecco's phosphate buffer saline (DPBS), trypan blue solution cell culture, dimethylsulfoxide (DMSO), isopropanol, Tris base, urea, HCL, ammonium biocarbonate, acetonitrile, dithiotheritol (DTT), iodoacetamine (IAA), formic acid, radio immunoprecipitation assay (RIPA) buffer, protease inhibitor cocktail and milli-Q water were all purchased from Sigma-Aldrich (Poole. UK). Mr. Frosty™ Freezing Container was purchased from ThermoFisher scientific (Waltham, MA, USA). Certified Sep-Pak C18 cc vac cartridge was purchased from (Waters, UK). Sequence grade modified trypsin purchased from Promega (Southampton, UK). Glutathione reductase, lactate dehydrogenase and lipid peroxidation (MDA) assay kits were purchased from Abcam (Cambridge, UK). Protein carbonyl colorimetric assay kit was purchased from Cayman Chemical Company (Ann Arbor, MI, USA).

Experimental design

The study was divided into three arms (**Figure 1**). **Arm 1** involved culturing HL-60 cells up to 70% confluence in RPMI 1460 media, containing 10% (v/v) FBS and 50 U/mL penicillin-streptomycin. HL-60 cells were centrifuged at 100 x g for 5 min and the medium was immediately removed. HL-60 cells were re-suspended in freezing media (10% DMSO and 90% FBS) at 10^6 cells/mL, slowly frozen in cryogenic tubes and stored at -80°C overnight. Next, cells were cryopreserved either in the freezing media in liquid nitrogen. HL-60 cells were thawed in a water bath at 37°C, centrifuged at 100 x g for 5 min and washed three times with RPMI media. Post thawing, HL-60 cells were cultured in a recovery medium containing RPMI, 20% FBS, 5 U/mL penicillin-streptomycin and the FBS concentration was reduced to 10% 24 h post thaw. HL-60 cells were cultured as described above for **Arm 1** with exception of adding 300 µM Nig (**Arm 2)** or 200 µM Sal (**Arm 3)** for 24 h prior to cryopreservation, during cryopreservation and up to 48 h post thaw. The selected concentrations of the cryo-additive agents (e.g.

Nig or Sal) were optimised as described in Supplement S1. Cells were maintained at all times in culture at 37°C under 5% $C0_2$/ 95% air.

For proteomic and biochemical analysis (n = 5 batches of cells per arm), HL-60 cells cryopreserved in DMSO +/- Nig or Sal were harvested at approximately 70% confluence prior to freezing and at 24h or 48 h post thaw.

Sample preparation for mass spectrometry

Extracted proteins from HL-60 cells prior to and 24 h post cryopreservation were precipitated by mixing cell pellets with cooled acetone. The cells were vortexed, incubated for 60 min at -20°C and centrifuged at 13,000 x g for 10 min. The supernatants were decanted and tubes were uncapped to let the acetone evaporate at room temperature for 30 min. Pelleted proteins were homogenised in 6 M urea buffer, vortexed and sonicated for 2 min. 70 mM DTT was added to samples and incubated 30-60 min at room temperature. Next 140 mM Iodoacetic acid alkylating reagent was added, followed by vortexing and incubation for 30-60 min at room temperature. The urea concentration was reduced by adding 775 milliQ water and vortexing. Protein concentrations were determined using the Bradford method. After this, 60 µg of extracted proteins were trypsinized in a 1:50 ratio, mixed carefully and left overnight at 37°C for digestion. The next day, the reactions were stopped via adjusting the pH to <6 by adding concentrated acetic acid. The digested peptides were purified using SEP-PAK C18 purification columns.

Nano-high-performance liquid chromatography-tandem mass spectrometry proteomic analyses

Qualitative and quantitative proteomic analyses were performed in a bi-dimensional microUPLC tandem nanoESI-HDMS^E platform by multiplexed data-independent acquisition experiments [27]. A 2D-RP/RP Acquity UPLC M-Class System (Waters Corporation: Milford, MA) coupled to a Synapt G2-Si mass spectrometer (Waters Corporation) platform was used. The samples were fractionated using a one-dimension reversed-phase approach. Peptide samples (0.5 µg) were loaded into a 100 Å, 1,8µm, 75 µm × 150 mm M-Class HSS T3 column (Waters Corporation). The fractionation was achieved by using an acetonitrile gradient from 7% to 40% (v/v) over 95 min at a flow rate of 0.4 µL/min directly

into a Synapt G2-Si mass spectrometer. For every measurement, the mass spectrometer was operated in resolution mode with an m/z resolving power of about 40,000 FWHM, using ion mobility with a cross-section resolving power of at least 40 Ω /ΔΩ. MS and MS/MS data were acquired in positive ion mode using ion mobility separation of precursor ions ($HDMS^E$) over a range of 50-2000 m/z. The lock mass channel was sampled every 30 s. The mass spectrometer was calibrated with a MS/MS spectrum of [Glu1]-fibrinopeptide B human (Glu-Fib) solution delivered through the reference sprayer of the NanoLock Spray source.

Validation assays

Enzymatic activities

HL-60 cell pellets were collected and washed in cold PBS once as described above and lysed in 350 µL RIPA buffer and 2.85 µL protease inhibitors and kept on ice for 30 min. Cell lysates were centrifuged at 100 x g for 5 min and enzymatic assays were performed using an amount equivalent to 1 x 10^6 HL-60 cells according to the manufacturer's instructions. The glutathione reductase (GR) assay is based on measuring spectrophotometrically the resulting chromophore (TNB) [e.g. sulfhydryl-glutathione and 5,5'-dithiobis (2-nitrobenzoic acid) (DNTB)] at 405 nm. The first and second readouts were measured at 5 and 10 min intervals using the Spectrostar Nano plate reader (Promega). Lactate dehydrogenase (LDH) assays were also performed according to the manufacturer's instructions. The quantity of NADH was detected spectrophotometrically at 450 nm by mixing NADH detection buffer with the cell supernatant and lysate. The first readout was taken immediately and the samples were incubated in the dark at 37°C with a final colorimetric reading at 30 min.

Protein and lipid oxidation assays

Protein oxidation or carbonylation was measured in two sets of samples (each sample is composed of 3 sets of HL-60 cells pooled together) prior to cryopreservation and 24 h post thaw. The carbonylation assay was performed according to the manufacturer's instructions. Briefly, a reaction between 2,4-dinitrophenylhydrazine (DNPH) and oxidized carbonyl groups on proteins was conducted using Cayman's protein assay kit. The derivatized carbonyl groups were quantitated by reading spectrophotometrically at 375 nm. For lipid peroxidation, measurements were carried out in triplicate

on amounts equivalent to 10^6 cells/mL by identifying the formation of malondialdehyde-thiobarbituric acid (MDA-TBA) adduct in acidic condition at 95°C for 1 h. Samples absorbance's were measured at 532 nm using the Spectrostar nano plate reader following the manufacturer's instructions. Malondialdehyde (MDA) concentration was expressed in nmol.

Cell proliferation

HL-60 cell viability and proliferation were assessed at1 h, 24 h and 48 h post thaw. Cells were mixed with trypan blue and placed on haemocytometer slides for counting under light microscope in duplicate at each time point.

Statistical analysis

All enzymatic assays were performed using five biological replicates. The lipid oxidation assay was performed in triplicate and the protein carbonylation assay was carried out in duplicate. Results were presented as mean ± standard deviation. Significant differences between groups were determined using Student's t-test for paired and unpaired observations. *P* values <0.05 were considered significant.

Availability of data materials

The mass spectrometry proteomics data have been deposited to the ProteomeXchange Consortium via the PRIDE partner repository with the dataset identifier PXD006998.

Abbreviations

ANOVA: Analysis of variance

CPAs: Cryo-protective agents

DMSO: Dimethylsulfoxide

DNPH: Dinitrophenylhydrazine

DTT: Dithiotheritol

DPBS: Dulbecco's phosphate buffer saline

FDR: False discovery rate

FBS: Fetal Bovine Serum

FC: Fold Changes

Glu-Fib: Glu1-fibrinopeptide B human

GR: Glutathione reductase

HL-60: Human Leukaemia cells

LDH: Lactate dehydrogenase

MDA: Malondialdehyde

MDA-TBA: Malondialdehyde-thiobarbituric acid

Nig: Nigerose

ND: Not Detected

PT: Post thaw

PC: Prior cryopreservation

RIPA: Radio immunoprecipitation assay

Sal: Salidroside

DNTB: Sulfhydryl-glutathione and 5, 5'-dithiobis [2-nitrobenzoic acid]

UP: Unique peptides

Declarations

Ethics approval and consent to participate

Not applicable.

Consent for publication

Not applicable

Competing interests

The authors declare no competing interests.

Funding

This work was supported by the King AbdulAziz City for Science and Technology research fund. JSC and DMS are funded by FAPESP (São Paulo Research Foundation, grants 2014/14881-1, 2013/08711-3 and 2014/10068-4) and CNPq (The Brazilian National Council for Scientific and Technological Development, grant 460289/2014-4).

Author contributions

NASA performed all experimental manipulations, sample preparation for mass spectrometry and prepared the tables and figures and performed bioinformatic analysis. JSC performed sample acquisition sample acquisition and data analysis mass spectrometry. DM supervised the proteomics pipeline. NKHS co-supervised the project. HR designed and supervised the project, performed biological interpretation of the data. NASA, JSC, DM, NKHS and HR wrote the manuscript. All authors edited otherwise approved the final version of the manuscript**.**

References

1. Valeri CR, Ragno G, Pivacek LE, Cassidy GP, Srey R, Hansson-Wicher M, Leavy ME. An Experiment with Glycerol-Frozen Red Blood Cells Stored at -80°C for up to 37 years. Vox Sanguinis 2000; 79(3):168–174.

2. Manson C, Brindley DA, Culme-Seymour EJ, Davie NL. Cell therapy industry: billion dollar global business with unlimited potential. Regen. Med. 2011; (6):265-272.

3. Beirão J, Zilli L, Vilella S, Cabrita E, Schiavone R et al. Improving sperm cryopreservation with antifreeze proteins: effect on gilthead seabream (Sparus aurata) plasma membrane lipids. Biol Reprod. 2012; 86 (2):59, 1-9

4. Alotaibi NAS, Slater, NKH, Rahmoune H. Salidroside as a Novel Protective Agent to Improve Red Blood Cell Cryopreservation. PLOS ONE 2016; 11(9), e0162748.

5. Hunt, C. J. Cryopreservation of Human Stem Cells for Clinical Application: A Review. Transfus. Med. Hemother. 2011; (38):107-123.

6. Fuller BJ. Cryoprotectants: the essential antifreezes to protect life in the frozen state. Cryo letters 2004; 25 (6), 375–88. Retrieved from http://www.ncbi.nlm.nih.gov/pubmed/15660165

7. Tatone C, Di Emidio G, Vento elena M, Artini PG. Cryopreservation and oxidative stress in reproductive cells. Gynecological Endocrinology 2010; 26(8):563-567.

8. Xu X, Cowley S, Flaim, CJ, James W, Seymour L, Cui, Z. The roles of apoptotic pathways in the low recovery rate after cryopreservation of dissociated human embryonic stem cells. Biotech Prog. 2010; 26(3):827–837.

9. Mathias FJ, D'Souza F, Uppangala S, Salian SR, Kalthur G, Adiga SK. Ovarian tissue vitrification is more efficient than slow freezing in protecting oocyte and granulosa cell DNA integrity. System Biology in Reprotductive Medicine 2014; 60(6):317-322.

10. Peris SI, Bilodeau JF, Dufour M, Bailey JL. Impact of cryopreservation and reactive oxygen species on DNA integrity, lipid peroxidation, and functional parameters in ram sperm. Molecular Reproductive and Development 2007; 74:878-892.

11. Chen GQ, Ren L, Zhang J, Reed BM, Zhang D, Shen XH. Cryopreservation affects ROS-induced oxidative stress and antioxidant response in Arabidopsis seedlings. Cryobiology 2015; 70(1):38-47.

12. Bagchi A, Woods EJ, Crister JK. Cryopreservation and vitrification: recent advances in fertility preservation technologies. Expert Rev Med Devices 2008; 5(3):359-370.

13. Wang S, Wang W, Xu Y, Tang M, Fang J, Sun H. et al. Proteomic characteristics of human sperm cryopreservation. Proteomics 2014; 14(2-3): 298–310.

14. Baumber J, Ball B, Linfor JJ. Assessment of cryopreservation of equine spermatozoa in the presence of enzyme scavengers and antioxidants. American Journal of Veterniary Research 2005; 66(5):772-779.

15. Stéphenne X, Najimi M, Sokal E. Hepatocyte cryopreservation: Is it time to change the strategy? World Journal of Gastroenterology 2010; 16(1), 1-14.

16. Nynca J, Arnold GJ, Frohlich T, Ciereszko A. Cryopreservation-induced alterations in protein composition of rainbow trout semen. Proteomics 2015; 15(15):2643-2654.

17. Sung JY, Md Saidur R, Woo SK, Do YR, Yoo JP, Myung GP. Proteomic identification of cryostress in epididymal spermatozoa. J Anim Sci Biotechnol. 2015; 7 (67):1-12.

18. Volk, G. M. Application of Functional Genomics and Proteomics to Plant Cryopreservation. Current Genomics 2010; 11(1):24-29.

19. Wagh V, Meganathan K, Hatap S, Gaspar JA, Winkler J, Spitkovsky D et al. Effects of cryopreservation on the transcriptome of human embryonic stem cells after thawing and culturing. Stem Cell Rev. 2011; 7(3):506-517.

20. Yoon SJ., Rahman MS, Kwon WS, Park YJ, Pang MG. Addition of Cryoprotectant Significantly Alters the Epididymal Sperm Proteome. PLoS ONE 2016; 11(3):e0152690.

21. Zilli L, Beirao J, Schiavone R, Herraez MP, Gnoni A, Vilella S. Comparative Proteome Analysis of Cryopreserved Flagella and Head Plasma Membrane Proteins from Sea Bream Spermatozoa: Effect of Antifreeze Proteins. PLOS ONE 2014; (6):e99992.

22. Kelly G. Rhodilla rosea: a possible plant adatogen. Alter Med Rev. 2001; 3:293-302.

23. Qian EW, Ge DT, Kong, SK. Salidroside protects human erythrocytes against hydrogen peroxide-induced apoptosis. Journal of Natural Products 2012; 75(4):531–537.

24. Mastuda K, Watanabe H, Fujimoto K, Aso K. Isolation of Nigerose and Kojibiose from Dextrans. Nature 1961; 191:278,

25. Consonni R, Cagliani LR, Cogliati C. NMR Characterization of Saccharides in Italian Honeys of Different Floral Sources. J. Agric. Food Chem. 2012; 60 (18):4526-4534.

26. Brandao-Teles C, Martins-de-Souza D, Guest PC, Cassoli JS. MK-801-Treated Oligodendrocytes as a Cellular Model to Study Schizophrenia. Advances in experimental medicine and biology 2017; 974:269-277.

27. Silva JC, Gorenstein MV, Li GZ, Vissers JP, Geromanos SJ. Absolute quantification of proteins by LCMSE: a virtue of parallel MS acquisition. Mol Cell Proteomics 2006; 5 (1):144-156.

28. Pathan M, et al. FunRich: An open access standalone functional enrichment and interaction network analysis tool. Proteomics 2015; 15:2597-2601.

29. Fahy G.M. Cryoprotectant toxicity: biochemical or osmotic? Cryo Letters 1984; 5:79–90.

30. Geiger T, Wehner A, Schaab C, Cox J, Mann M. Comparative proteomic analysis of eleven common cell lines reveals ubiquitous but varying expression of most proteins. Mol Cell Proteomics 2012; 11(3):M111.014050.

31. Marcucci F, Corti A, Berenson R. Ways to improve tumour uptake and penetration of drugs into solid tumors. Forntiers Research Topics 2010; 3:1-14.

32. Herberth M, Koethe D, Cheng T, Krzyszton ND, Schoeffmann S, Guest PC et al. Impaired glycolytic response in peripheral blood mononuclear cells of first-onset antipsychotic-naive schizophrenia patients. Mol Psychiatry 2011; 16(8):848-859.

33. Lunt SY, Vander Heiden MG. Aerobic glycolysis: meeting the metabolic requirements of cell proliferation. Annu. Rev. Cell Dev. Biol. 2011; 27:441–464.

34. Baust JG,Gao D, Baust, JM. Cryopreservation: An emerging paradigm change. Organogenesis 2009; 5(3):90–96.

35. Iuso A, Scacco S, Piccoli C, Bellomo F, Petruzzella V, Trentadue R et al. Dysfunctions of cellular oxidative metabolism in patients with mutations in the NDUFS1 and NDUFS4 genes of complex I. Biol . 2006; 281(15):10374-10380.

36. Ricci J, Munoz-Pinedo C, Fitzgerald P, Bailly-Maitre B, Perkins G, Yadava N et al. Disruption of mitochondrial function during apoptosis is mediated by caspase cleavage of the p75 subunit of complex I of the electron transport chain. Cell 2004; 117(6):773-786.

37. Glanemann C, Loos A, Gorret N et al. Disparity between changes in mRNA abundance and enzyme activity in Corynebacterium glutamicum: implications for DNA microarray analysis. Appl Microbiol Biotechnol. 2003; 61:61–68.

38. Mayer M. Hsp70 chaperone dynamics and molecular mechanism. Trends in Biochem Sci. 2013; 38(10):507-514.

39. Xue Li, Ozlem E, Liang L, Qidong Y, Andrew W, Wei D. Binding to WGR Domain by Salidroside Activates PARP1 and Protects Hematopoietic Stem Cells from Oxidative Stress. Antioxid Redox Signal 2014; 20(12):1853–1865.

40. Oda K, Matsuoka Y, Funahashi A, Kitano H. A comprehensive pathway map of epidermal growth factor receptor signaling. Mol Syst Biol. 2005; doi: 10.1038/msb4100014.

41. Lou Z, Chini C, Minter-Dykhouse K, Chen J. Mediator of DNA damage checkpoint protein 1 regulates BRCA1 localization and phosphorylation in DNA damage checkpoint control. J Biol Chem. 2003; 278:13599-13602.

42. Cui R, Zhanf H, Guo X, Cui Q, Wang J, Dai J. Proteomic analysis of cell proliferation in a human hepatic cell line (HL-7702) induced by perfluorooctane sulfonate using iTRAQ. J Hazard Mater. 2015; 299:361-370.

43. Hu X, Zhang X, Qiu S, Yu D, Lin S. Salidroside induces cell-cycle arrest and apoptosis in breast cancer. Biochemcial and Biophysical Research Communications 2011; 398(1):62-67.

44. Qi YJ, Cui S, Lu D, Yang YZ, Luo Y, Ma L et al. Effects of the aqueous extract of a Tibetan herb, Rhodiola algida vartangutica on proliferation and HIF-1 alfa, HIF-2 alfa expression in MCF-7 cells under hypoxic condition in vitro. Cancer Cell Int. 2015; 15(81):1-9.

Figure. 1. Schematic diagram. Experimental design of HL-60 cryopreserved in Dimethylsulfoxide (DMSO) [n=5] +/- Nigerose (Nig) [n=5] or Salidroside (Sal) [n=5]. Proteomic analysis and corresponding biological assays were conducted 24 h prior and post cryopreservation of HL-60 cell cultures grown in RPMI-1640 media (RPMI) +/- Nig or Sal.

Figure. 2. **Proteome analysis**. HL-60 total number of differentially expressed proteins cryopreserved in DMSO +/- Nig or Sal [n=5 per arm]. **A)** Venn diagram illustrating HL-60 cells unique and overlapped number of significantly changing proteins 24 h prior and post thaw. The numbers in the circles represent the number of identified genes significantly changing prior/post HL-60 cryopreserved in DMSO only [n=5], DMSO + Nig [n=5] or DMSO + Sal [n=5]. **B)** Table representing the total number of number of identified genes representing HL-60 upregulated (blue arrow) and downregulated (red arrow) proteins in each of the above cryo-condition.

Figure. 3. Biological pathways analysis. Comparative overview of the biological processes (**A**) and functional functions (**B**) representing mammalian HL-60 cells cryopreserved in DMSO +/- Nig or Sal. The percentage of proteins extracted from HL-60 cells cryopreserved in DMSO alone, DMSO/Nig or DMSO/Sal were identified using FunRich software.

Figure 4. Cell growth. HL-60 cell proliferation was measured in duplicate at 1h, 24 h and 48 h post thaw. Cells were initially either cultured in RPMI media containing Nig (300 µM) or Sal (200 µM) and cryopreserved in DMSO +/- Nig or Sal. HL-60 cells were thawed and cultured in RPMI media containing Nig (300 µM) or Sal (200 µM) for up to 48 h. Data are expressed as mean.

Figure 5. Oxido-Redox enzymatic assays. Intra-cellular enzymatic activities of HL-60 were measured prior freezing (Control). Cells were frozen in DMSO +/- Sal or Nig and HL-60 GR and LDH activities were measured in RPMI media only, RPMI +Nig (300 µM) or in RPMI + Sal (200 µM) 24 h post thaw. **A)** Glutathione reductase (GR) activity (mU/ml). **B)** LDH activity (mU/ml). Data are presented as a mean [n=5] ± SD. (* P value < 0.05).

Figure 6. Lipid peroxidation (MDA) assay. Lipid oxidation of HL-60 incubated prior and post thaw in media +/- Nig or Sal and cryopreserved in DMSO +/- Nig (300 µM) or Sal (200 µM). The data are represented in mean [n=3] ± SD (* P value <0.05).

Figure 7. Protein carbonylation of cryopreserved HL-60 cells. The control represents protein carbonylation level prior HL-60 cryopreservation in RPMI only, RPMI + 300 µM Nig or RPMI + 200 µM Sal. Cells were cryopreserved in RPMI/DMSO +/- Nig or Sal and protein carbonylation was measured in duplicate (each sample is composed of 3 sets of HL-60 cells pooled together) 1 h post thaw in RPMI media containing Sal or Nig. Data are expressed as mean ± SD (* P value <0.05).

Figure S1. CPAs dose response. The effect of Nig and Sal at different concentrations on HL-60 cell viability post cryopreservation in 10% DMSO +/- Nig or Sal. HL-60 cell cryosurvival was measured in triplicate using trypan blue.

Table 1: Proteins found at significantly different levels (p<0.05) using label-free LCMS/MS profiling of the human promyelocytic leukemia HL-60 cells cryopreserved in DMSO [n=5] +/- Sal [n=5] or Nig [n=5].

Uniprot entry	Protein name	DMSO alone UP	DMSO alone FC (PC/PT)	DMSO/nigerose UP	DMSO/nigerose FC (PC/PT)	DMSO/salidroside UP	DMSO/salidroside FC (PC/PT)
	**Oxido-Redox**						
Q99497	Protein deglycase DJ-1	ND		ND		12	1.4
P00338	Lactate dehydrogenase chain A	11	-1.6	ND		11	-1.6
P00390	Glutathione reductase	7	3.2	ND		ND	
P00441	Superoxide dismutase [Cu-Zn]	8	1.4	ND		ND	
Q16881	Thioredoxin reductase 1	2	14.6	2	35.0	2	15
P28331	NADH-ubiquinone oxidoreductase 75 kDa subunit	4	4.9	4	46.0	4	16
Q9Y2Q3	Glutathione S-transferase kappa 1	2	-8.0	2	-13.6	2	-3.5
P30048	Thioredoxin-dependent peroxide reductase, mitochondrial	2	-3.0	2	-5.2	2	-8.8
C9J0G0	Acyl-coenzyme A oxidase (ACOX)	2	32.0	2	16.8	2	42.7
P49748	Very long-chain specific acyl-CoA dehydrogenase	5	-2.7	5	-11.6	5	-14.8
P16152	Carbonyl reductase	ND		ND		5	-1.5
P49368	T-complex protein 1 subunit gamma	ND		17	1.2	ND	
P40227	T-complex protein 1 subunit zeta	ND		7	1.4	ND	
Q9NZL4	Hsp70-binding protein 1	3	14.4	3	-71	3	-77.0
P48723	Heat shock 70 kDa protein 13	ND		2	15.8	ND	
P34932	Heat shock 70 kDa protein 4	ND		ND		17	1.3
Q53EL6	Programmed cell death protein 4	ND		ND		4	1.6
P08758	Annexin A5 (Annexin-V)	6	-6.6	6	-9.2	6	4.5
Q5VT06	Centrosome-associated protein 350	29	88.9	29	61.2	29	81.2
P25787	Proteomsome subunit alpha type-2 (PSAT2	ND		3	34.4	ND	
	**Nuclear activities regulation**						
Q9BTE3	Mini-chromosome maintenance complex-binding protein	ND		2	11.0	2	70.0
P33993	DNA replication licensing factor MCM7	ND		ND		9	2.4
P35658	Nuclear pore complex protein Nup214	ND		ND		6	1.6
Q86YP4	Transcriptional repressor p66-alpha	ND		ND		11	2.5
Q5T890	DNA excision repair protein ERCC-6-like	4	-14.4	ND		4	8.8
Q99973	Telomerase protein component 1	ND		3	-2.3	3	-2.3
Q8WXI9	Transcriptional repressor p66-beta	4	-2.6	ND		ND	
O14980	Exportin-1	5	3.0	ND		5	3.7

A6H8Y1	Transcription factor TFIIIB component B	9	2.1	9	7.9	9	10.6
Q15054	DNA polymerase delta subunit 3	2	3.4	2	30.0	2	22.3
	**Cell growth and function**						
P00533	Epidermal growth factor receptor	ND		ND		4	2.1
Q14676	Mediator of DNA damage checkpoint protein 1	ND		5	17.0	5	21.4
Q6ZUM4	Rho GTPase-activating protein 27	2	13.7	2	39.5	2	75.4
Q9BYX2	TBC1 domain family member 2A	ND		3	11.2	3	39.0
O14976	Cyclin-G-associated kinase	4	4.1	4	8.5	4	9.8
Q8N163	Cell cycle and apoptosis regulator protein 2	ND		9	1.8	9	2.3
O94986	Centrosomal protein 152 KDa	ND		7	59.8	7	19.0
Q13576	RasGTPase-activating-like protein IQGAP2	4	15.2	4	65.7	4	40.9
Q14789	Golgin subfamily B member	14	18.9	14	37.2	14	21.3
P49327	Fatty acid synthase	ND		39	10.4	39	9.0
Q01484	Ankyrin-2	17	32.0	17	39.7	17	48.8
O00423	Echinoderm microtubule-associated protein-like 1	4	23.0	4	42.0	4	32.2
A0A0U1RR07	Synaptotagmin-like protein 2	4	4.1	4	9.0	4	22.0
Q15691	Microtubule-associated protein RP/EB family member 1	10	7.1	10	3.2	10	7.1
E9PNZ4	Microtubule-actin cross-linking factor 1, isoforms 1/2/3/5	2	12.6	2	12.3	2	4.4

Abbreviations: UP = unique peptides, ND = Not Detected, FC = Fold Changes indicating the ratio of differentially expressed proteins identified prior cryopreservation (PC) and post thaw (PT).

Figure. 1

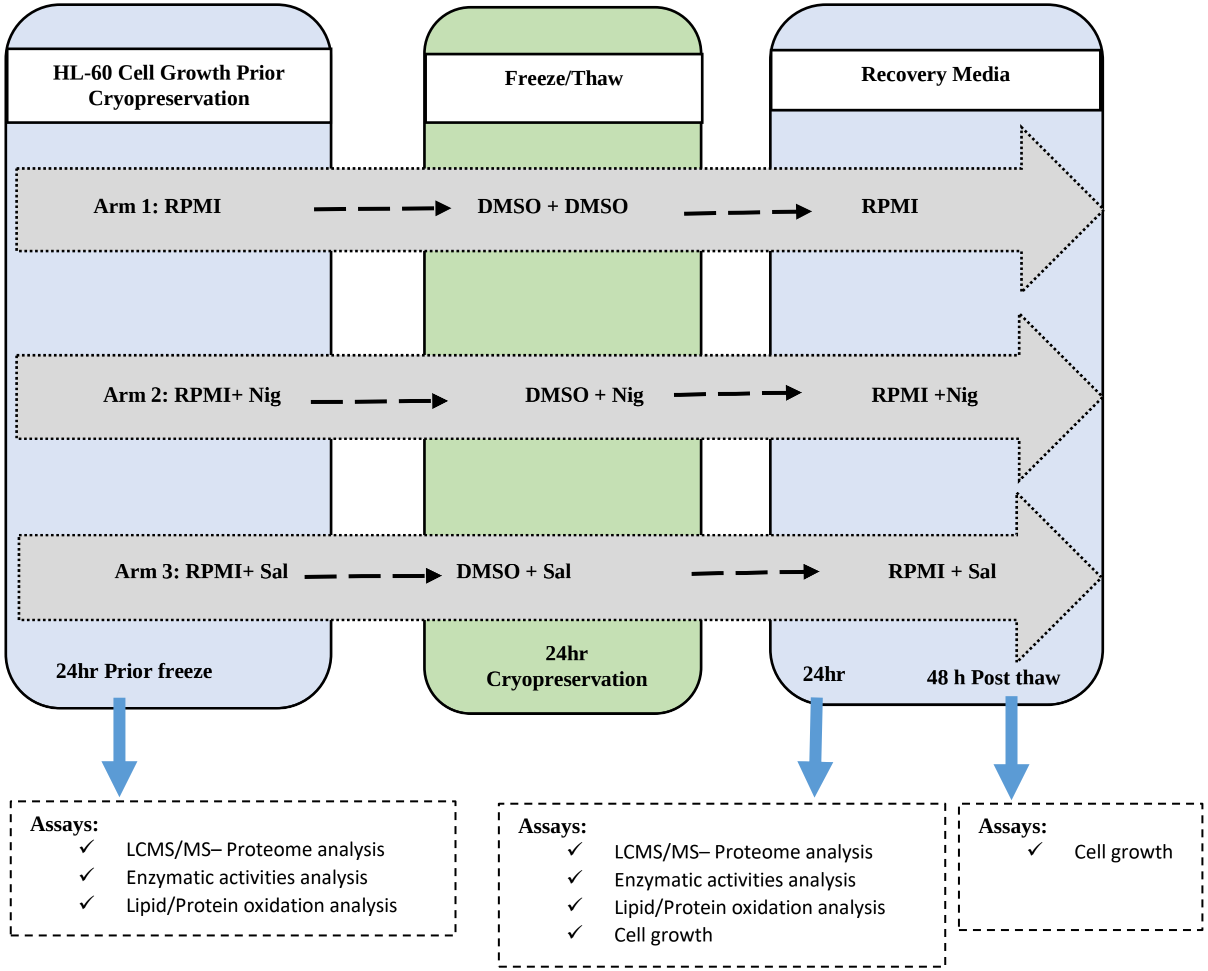
HL-60 Cell Growth Prior Cryopreservation
Freeze/Thaw
Recovery Media
Arm 1: RPMI
DMSO + DMSO
RPMI
Arm 2: RPMI+ Nig
DMSO + Nig
RPMI +Nig
Arm 3: RPMI+ Sal
DMSO + Sal
RPMI + Sal
24hr Prior freeze
24hr Cryopreservation
24hr
48 h Post thaw
Assays:
✓ LCMS/MS– Proteome analysis
✓ Enzymatic activities analysis
✓ Lipid/Protein oxidation analysis
Assays:
✓ LCMS/MS– Proteome analysis
✓ Enzymatic activities analysis
✓ Lipid/Protein oxidation analysis
✓ Cell growth
Assays:
✓ Cell growth

Figure 2.

A)

**Conditions**	**DMSO only**	**DMSO/Nig**	**DMSO/Sal**
No. of identified proteins	887	1140	1032
No. of identified genes	892	1152	1059

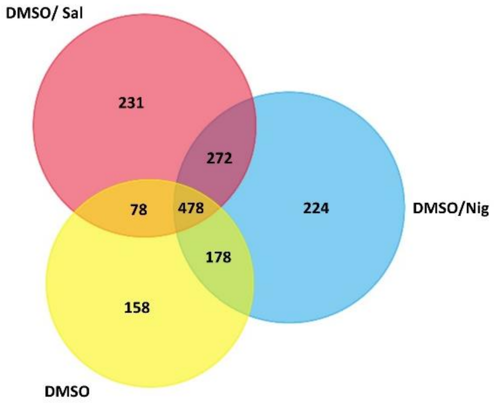

B)

**Up/down regulated**	**DMSO only**	**DMSO/Nig**	**DMSO/Sal**
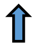	484	536	491
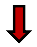	403	604	541

Figure 3.

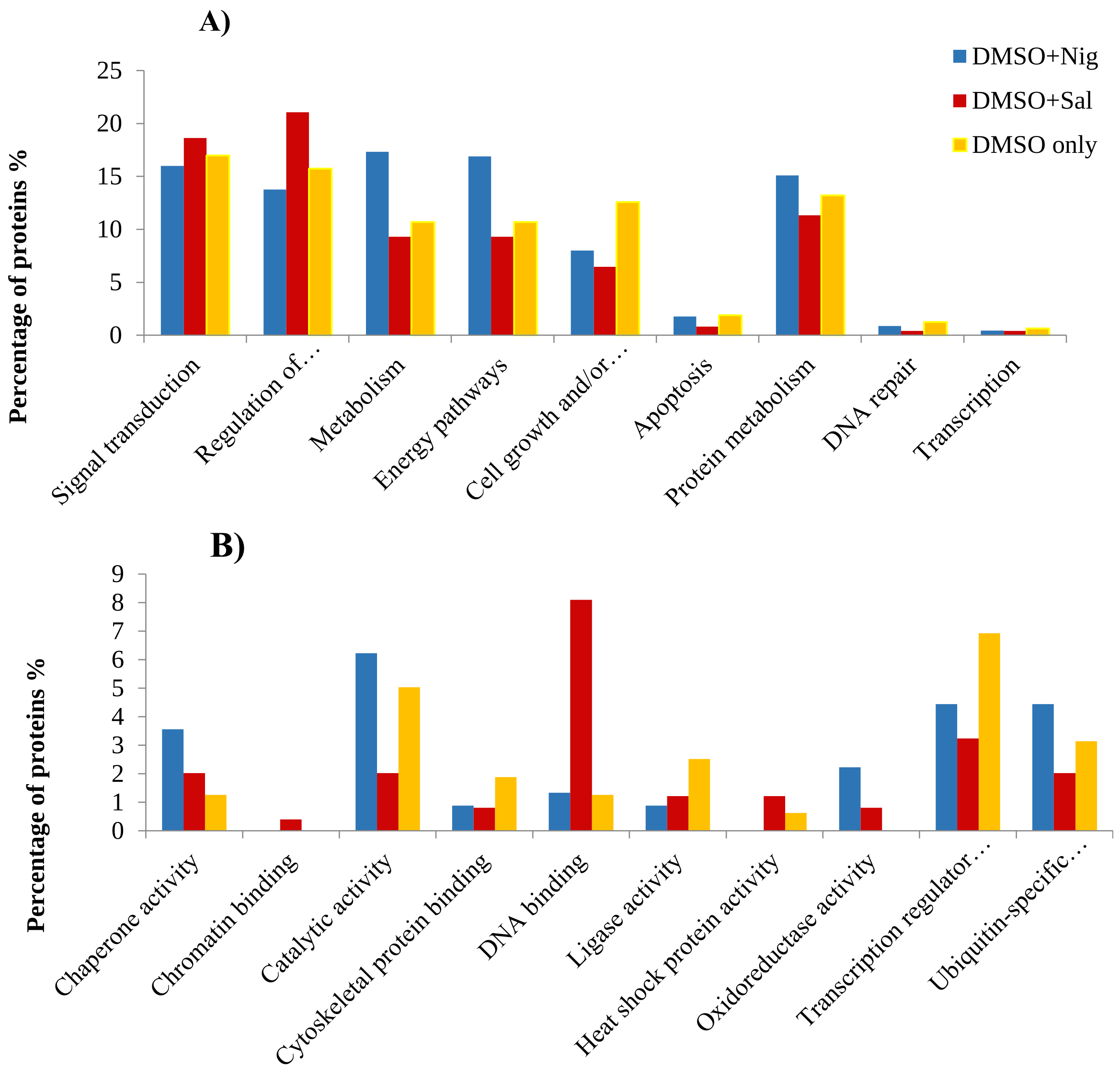
A)
DMSO+Nig
DMSO+Sal
DMSO only
Percentage of proteins %
25
20
15
10
5
0
Signal transduction
Regulation of…
Metabolism
Energy pathways
Cell growth and/or…
Apoptosis
Protein metabolism
DNA repair
Transcription
B)
Percentage of proteins %
9
8
7
6
5
4
3
2
1
0
Chaperone activity
Chromatin binding
Catalytic activity
Cytoskeletal protein binding
DNA binding
Ligase activity
Heat shock protein activity
Oxidoreductase activity
Transcription regulator…
Ubiquitin-specific…

Figure 4.

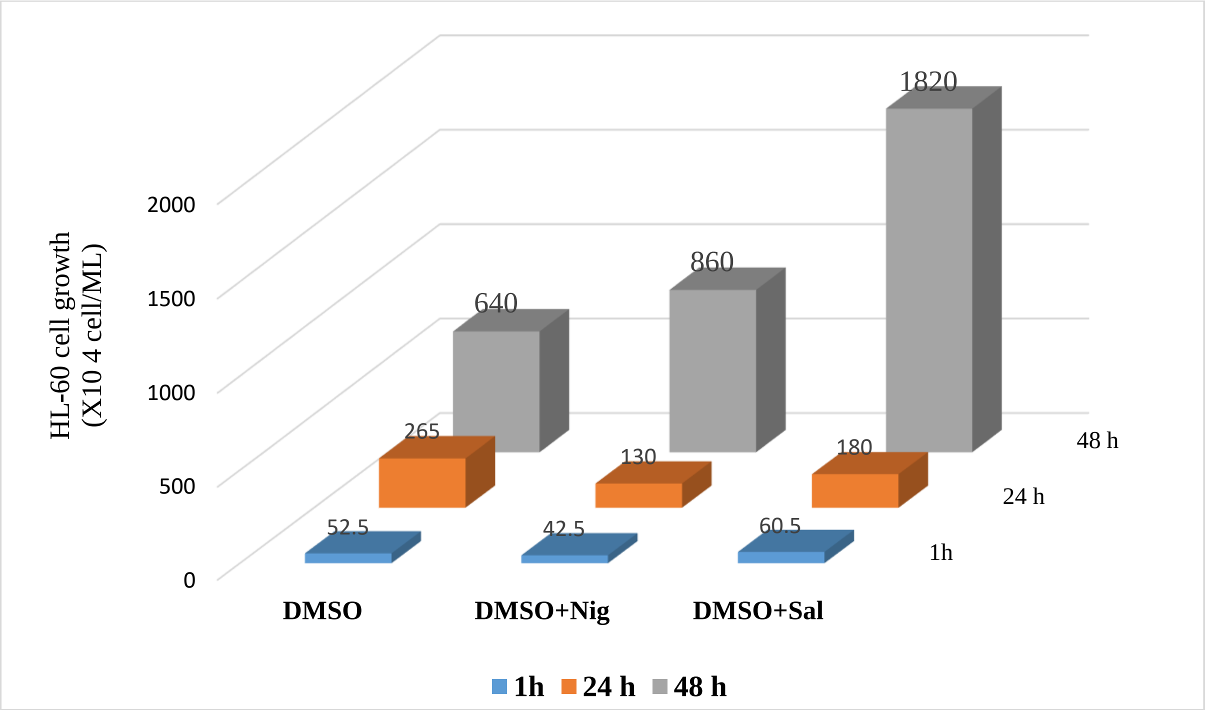
HL-60 cell growth
(X10 4 cell/ML)
2000
1500
1000
500
0
1820
860
640
265
130
180
52.5
42.5
60.5
48 h
24 h
1h
DMSO
DMSO+Nig
DMSO+Sal
1h
24 h
48 h

FIG.5

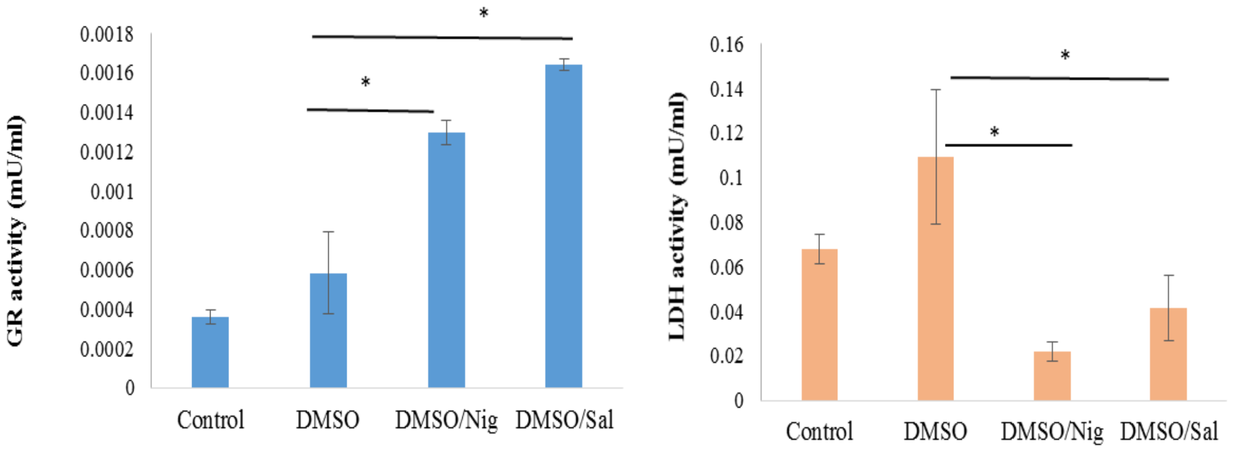

GR activity (mU/ml)
0.0018
0.0016
0.0014
0.0012
0.001
0.0008
0.0006
0.0004
0.0002
0
*
*
Control
DMSO
DMSO/Nig
DMSO/Sal
LDH activity (mU/ml)
0.16
0.14
0.12
0.1
0.08
0.06
0.04
0.02
0
*
*
Control
DMSO
DMSO/Nig
DMSO/Sal

Figure 6.

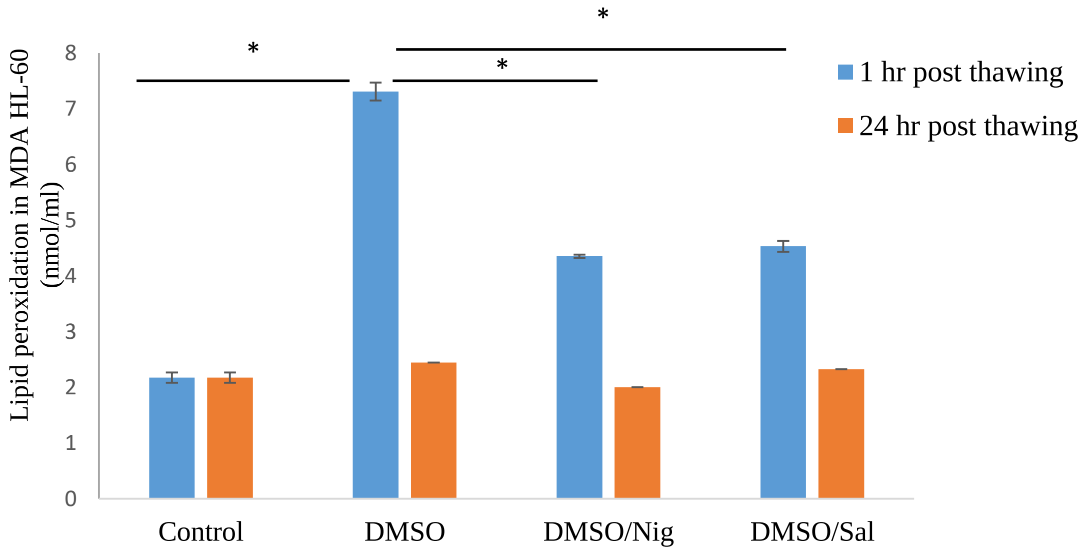
Lipid peroxidation in MDA HL-60 (nmol/ml)
0
1
2
3
4
5
6
7
8
*
*
*
1 hr post thawing
24 hr post thawing
Control
DMSO
DMSO/Nig
DMSO/Sal

Figure 7.

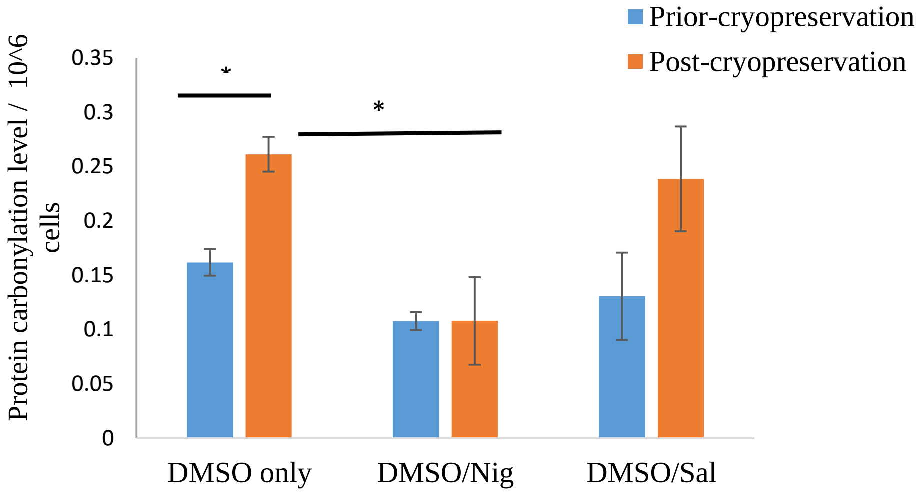
Prior-cryopreservation
Post-cryopreservation
Protein carbonylation level / 10^6 cells
0.35
0.3
0.25
0.2
0.15
0.1
0.05
0
*
*
DMSO only
DMSO/Nig
DMSO/Sal

Supplement S1

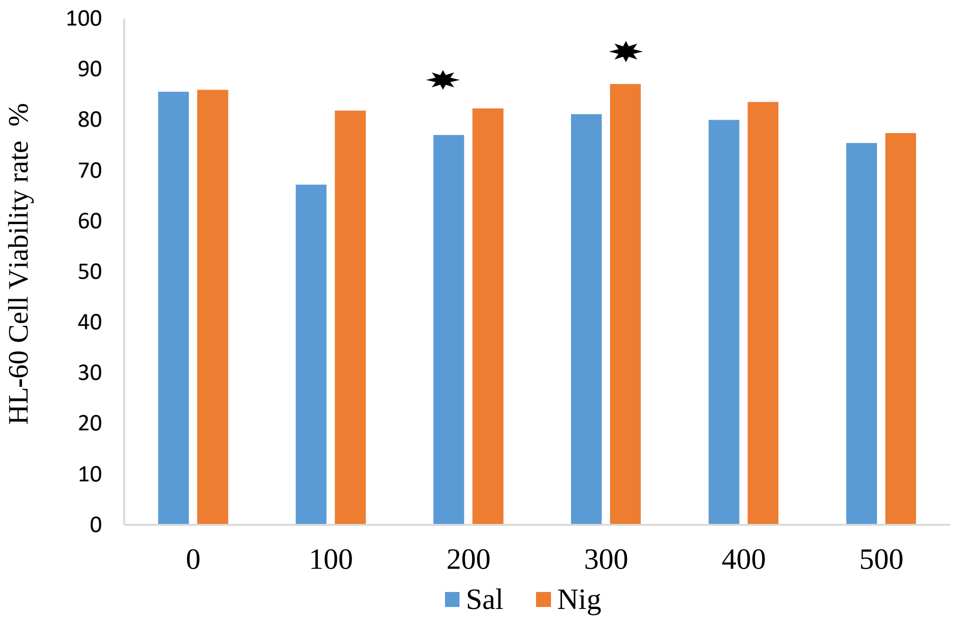

Click here to download Table Table 1 pdf..pdf

Uniprot entry	Protein name	DMSO alone UP	DMSO alone FC (PC/PT)	DMSO/nigerose UP	DMSO/nigerose FC (PC/PT)	DMSO/salidroside UP	DMSO/salidroside FC (PC/PT)
	**Oxido-Redox**						
Q99497	Protein deglycase DJ-1	ND		ND		12	1.4
P00338	Lactate dehydrogenase chain A	11	-1.6	ND		11	-1.6
P00390	Glutathione reductase	7	3.2	ND		ND	
P00441	Superoxide dismutase [Cu-Zn]	8	1.4	ND		ND	
Q16881	Thioredoxin reductase 1	2	14.6	2	35.0	2	15
P28331	NADH-ubiquinone oxidoreductase 75 kDa subunit	4	4.9	4	46.0	4	16
Q9Y2Q3	Glutathione S-transferase kappa 1	2	-8.0	2	-13.6	2	-3.5
P30048	Thioredoxin-dependent peroxide reductase, mitochondrial	2	-3.0	2	-5.2	2	-8.8
C9J0G0	Acyl-coenzyme A oxidase (ACOX)	2	32.0	2	16.8	2	42.7
P49748	Very long-chain specific acyl-CoA dehydrogenase	5	-2.7	5	-11.6	5	-14.8
P16152	Carbonyl reductase	ND		ND		5	-1.5
P49368	T-complex protein 1 subunit gamma	ND		17	1.2	ND	
P40227	T-complex protein 1 subunit zeta	ND		7	1.4	ND	
Q9NZL4	Hsp70-binding protein 1	3	14.4	3	-71	3	-77.0
P48723	Heat shock 70 kDa protein 13	ND		2	15.8	ND	
P34932	Heat shock 70 kDa protein 4	ND		ND		17	1.3
Q53EL6	Programmed cell death protein 4	ND		ND		4	1.6
P08758	Annexin A5 (Annexin-V)	6	-6.6	6	-9.2	6	4.5
Q5VT06	Centrosome-associated protein 350	29	88.9	29	61.2	29	81.2
P25787	Proteomsome subunit alpha type-2 (PSAT2	ND		3	34.4	ND	
	**Nuclear activities regulation**						
Q9BTE3	Mini-chromosome maintenance complex-binding protein	ND		2	11.0	2	70.0
P33993	DNA replication licensing factor MCM7	ND		ND		9	2.4
P35658	Nuclear pore complex protein Nup214	ND		ND		6	1.6
Q86YP4	Transcriptional repressor p66-alpha	ND		ND		11	2.5
Q5T890	DNA excision repair protein ERCC-6-like	4	-14.4	ND		4	8.8
Q99973	Telomerase protein component 1	ND		3	-2.3	3	-2.3
Q8WXI9	Transcriptional repressor p66-beta	4	-2.6	ND		ND	
O14980	Exportin-1	5	3.0	ND		5	3.7

A6H8Y1	Transcription factor TFIIIB component B	9	2.1	9	7.9	9	10.6
Q15054	DNA polymerase delta subunit 3	2	3.4	2	30.0	2	22.3
	**Cell growth and function**						
P00533	Epidermal growth factor receptor	ND		ND		4	2.1
Q14676	Mediator of DNA damage checkpoint protein 1	ND		5	17.0	5	21.4
Q6ZUM4	Rho GTPase-activating protein 27	2	13.7	2	39.5	2	75.4
Q9BYX2	TBC1 domain family member 2A	ND		3	11.2	3	39.0
O14976	Cyclin-G-associated kinase	4	4.1	4	8.5	4	9.8
Q8N163	Cell cycle and apoptosis regulator protein 2	ND		9	1.8	9	2.3
O94986	Centrosomal protein 152 KDa	ND		7	59.8	7	19.0
Q13576	RasGTPase-activating-like protein IQGAP2	4	15.2	4	65.7	4	40.9
Q14789	Golgin subfamily B member	14	18.9	14	37.2	14	21.3
P49327	Fatty acid synthase	ND		39	10.4	39	9.0
Q01484	Ankyrin-2	17	32.0	17	39.7	17	48.8
O00423	Echinoderm microtubule-associated protein-like 1	4	23.0	4	42.0	4	32.2
A0A0U1RR07	Synaptotagmin-like protein 2	4	4.1	4	9.0	4	22.0
Q15691	Microtubule-associated protein RP/EB family member 1	10	7.1	10	3.2	10	7.1
E9PNZ4	Microtubule-actin cross-linking factor 1, isoforms 1/2/3/5	2	12.6	2	12.3	2	4.4

Abbreviations: UP = unique peptides, ND = Not Detected, FC = Fold Changes indicating the ratio of differentially expressed proteins identified prior cryopreservation (PC) and post thaw (PT).

Figure. 1

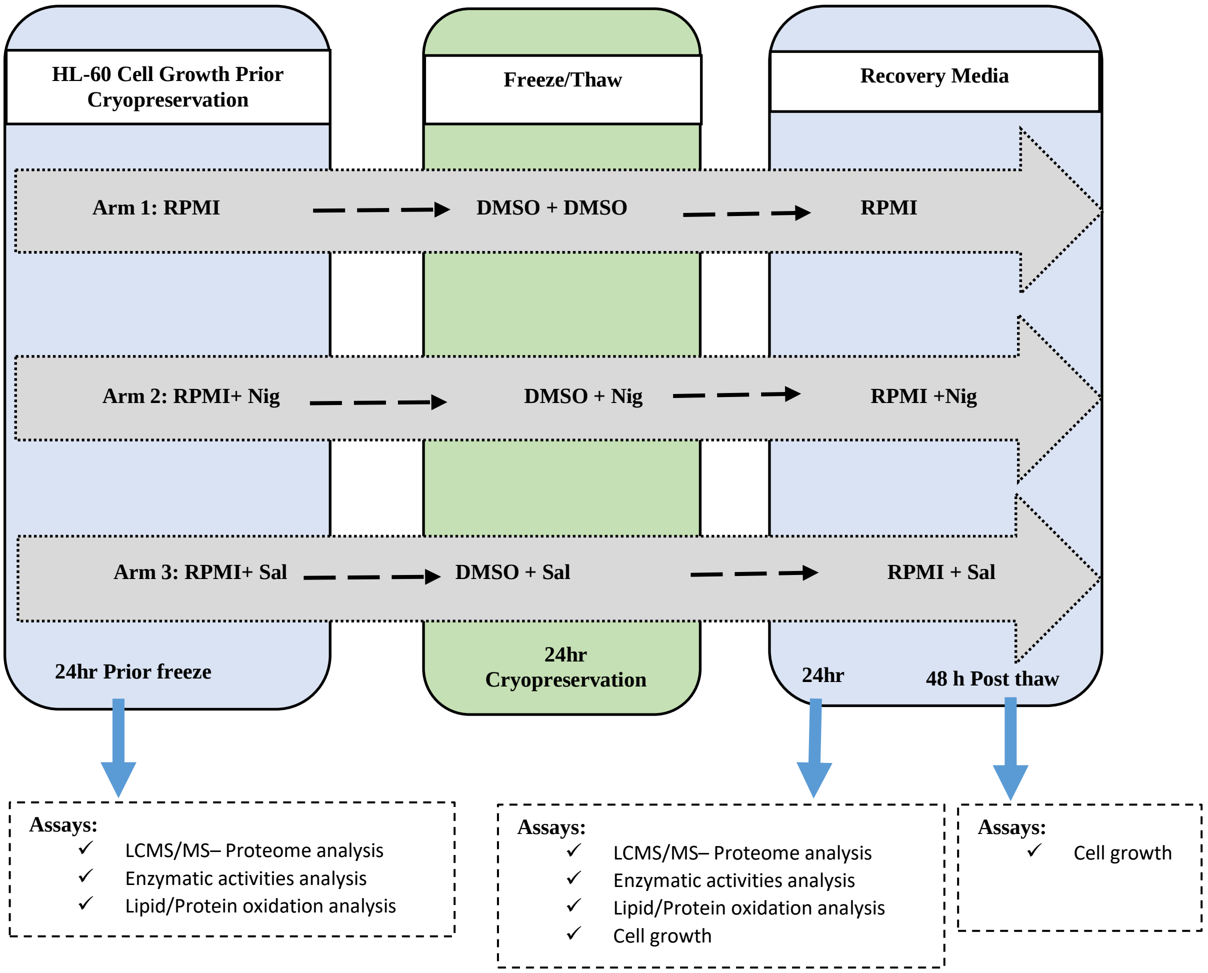
HL-60 Cell Growth Prior Cryopreservation
Freeze/Thaw
Recovery Media
Arm 1: RPMI
DMSO + DMSO
RPMI
Arm 2: RPMI+ Nig
DMSO + Nig
RPMI +Nig
Arm 3: RPMI+ Sal
DMSO + Sal
RPMI + Sal
24hr Prior freeze
24hr Cryopreservation
24hr
48 h Post thaw
Assays:
LCMS/MS– Proteome analysis
Enzymatic activities analysis
Lipid/Protein oxidation analysis
Assays:
LCMS/MS– Proteome analysis
Enzymatic activities analysis
Lipid/Protein oxidation analysis
Cell growth
Assays:
Cell growth

Figure 2 Click here to download Figure Figure 2 pdf..pdf

Figure 2.

A)

**Conditions**	**DMSO only**	**DMSO/Nig**	**DMSO/Sal**
No. of identified proteins	887	1140	1032
No. of identified genes	892	1152	1059

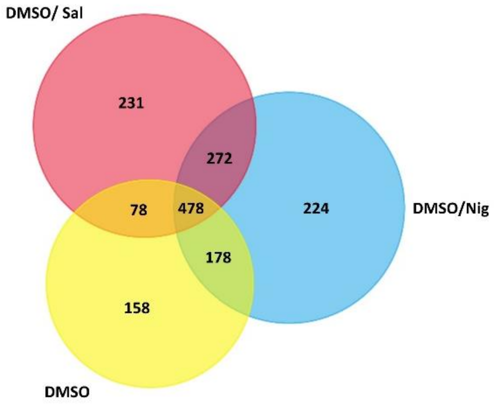

B)

**Up/down regulated**	**DMSO only**	**DMSO/Nig**	**DMSO/Sal**
↑	484	536	491
↓	403	604	541

Figure 3.

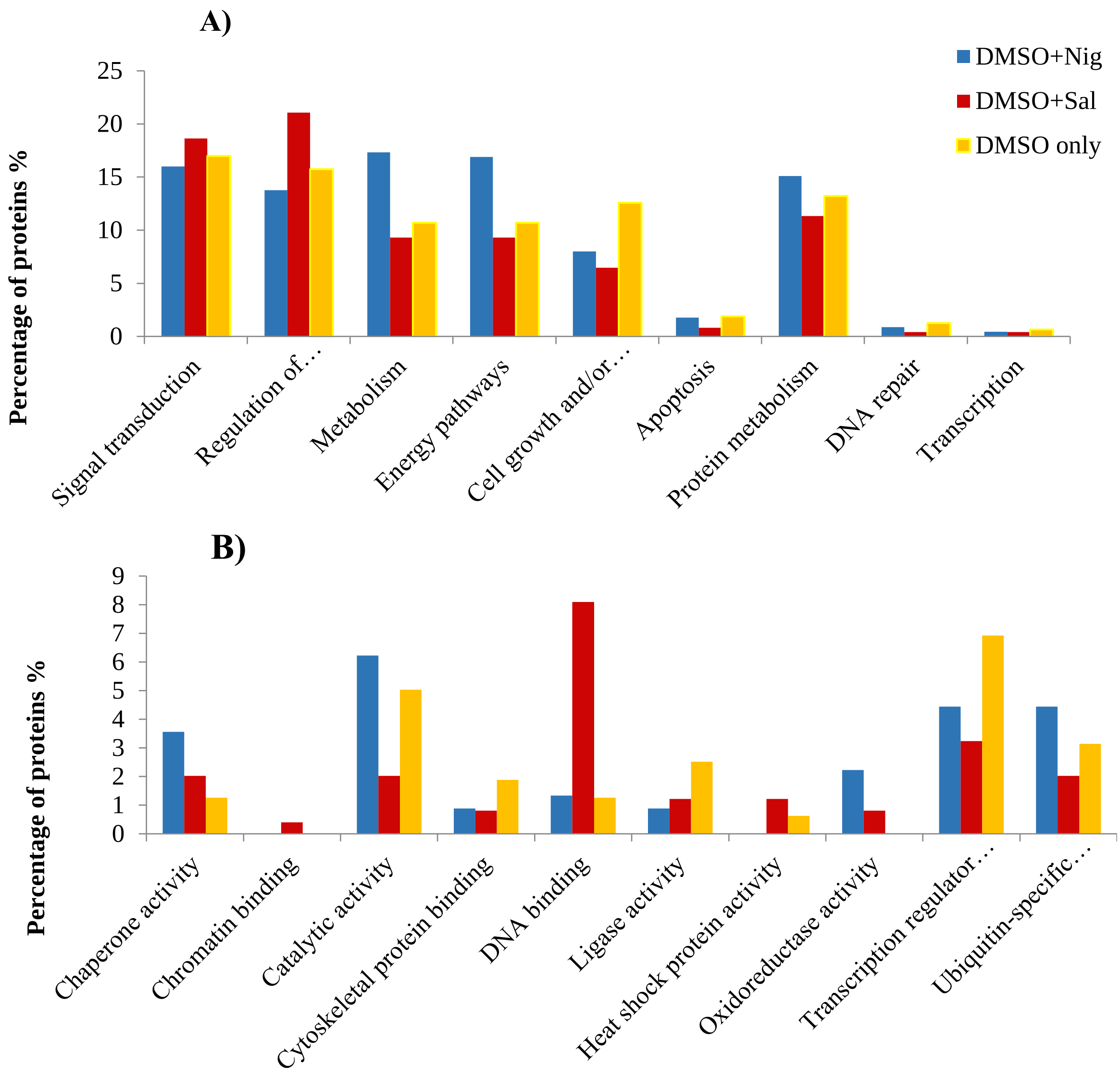
A)
DMSO+Nig
DMSO+Sal
DMSO only
Percentage of proteins %
25
20
15
10
5
0
Signal transduction
Regulation of…
Metabolism
Energy pathways
Cell growth and/or…
Apoptosis
Protein metabolism
DNA repair
Transcription
B)
Percentage of proteins %
9
8
7
6
5
4
3
2
1
0
Chaperone activity
Chromatin binding
Catalytic activity
Cytoskeletal protein binding
DNA binding
Ligase activity
Heat shock protein activity
Oxidoreductase activity
Transcription regulator…
Ubiquitin-specific…

Figure 4.

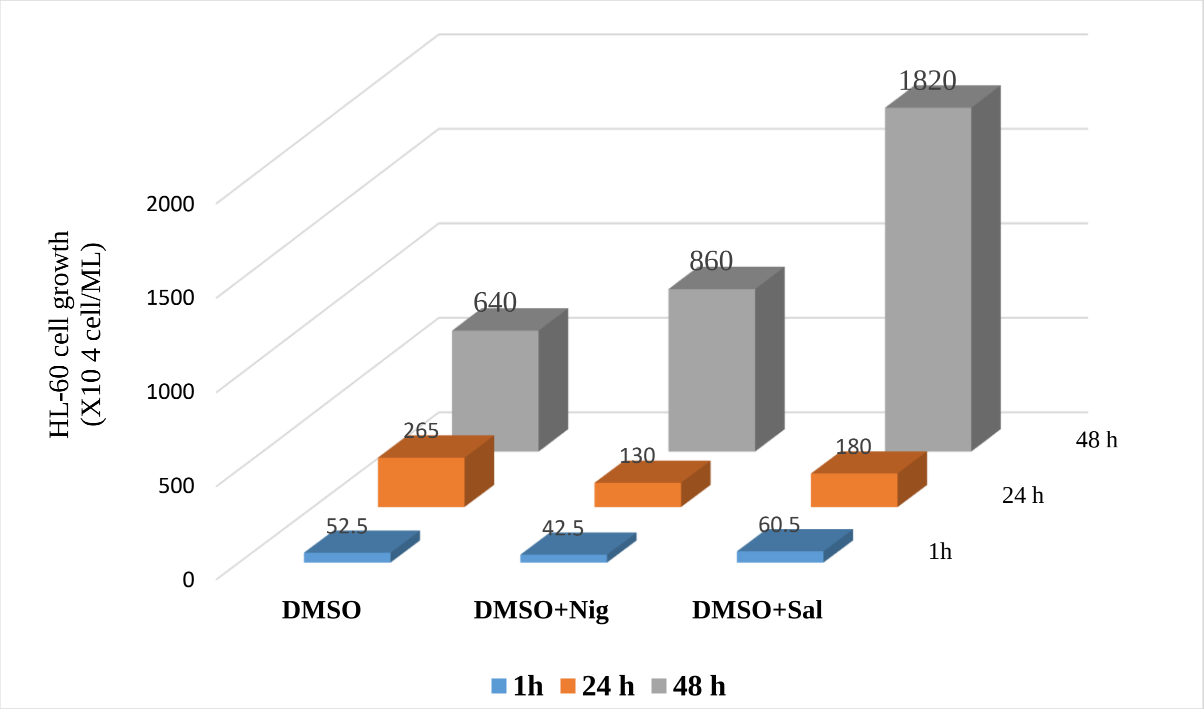

HL-60 cell growth
(X10 4 cell/ML)
0
500
1000
1500
2000
52.5
42.5
60.5
265
130
180
640
860
1820
1h
24 h
48 h
DMSO
DMSO+Nig
DMSO+Sal
1h 24 h 48 h

 Click here to download Figure Figure 5 pdf..pdf

Figure 5

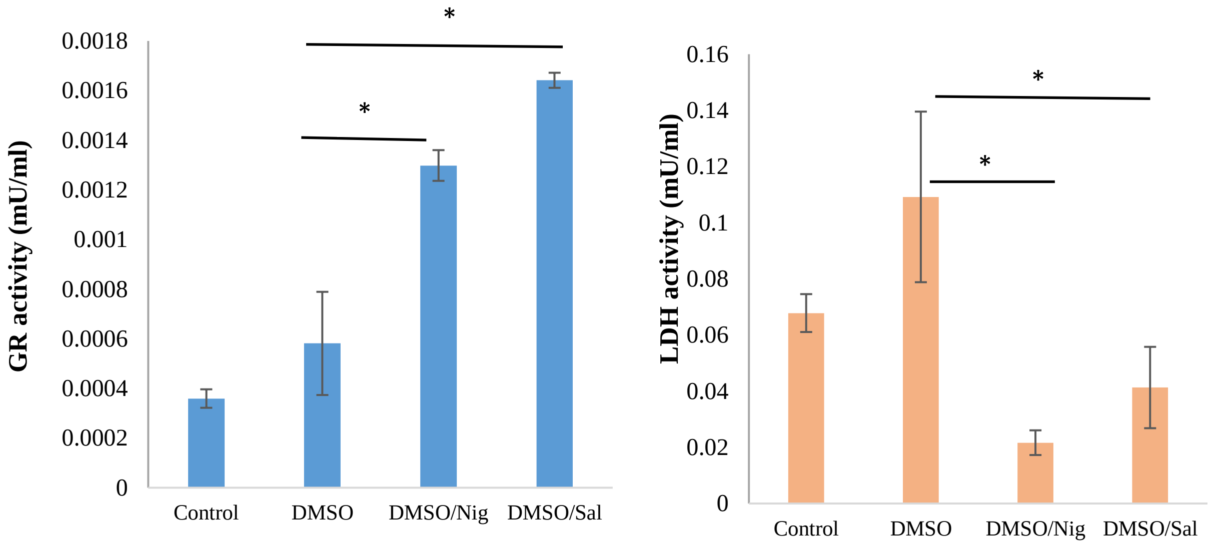

Figure 6 Click here to download Figure Figure 6 pdf..pdf

Figure 6.

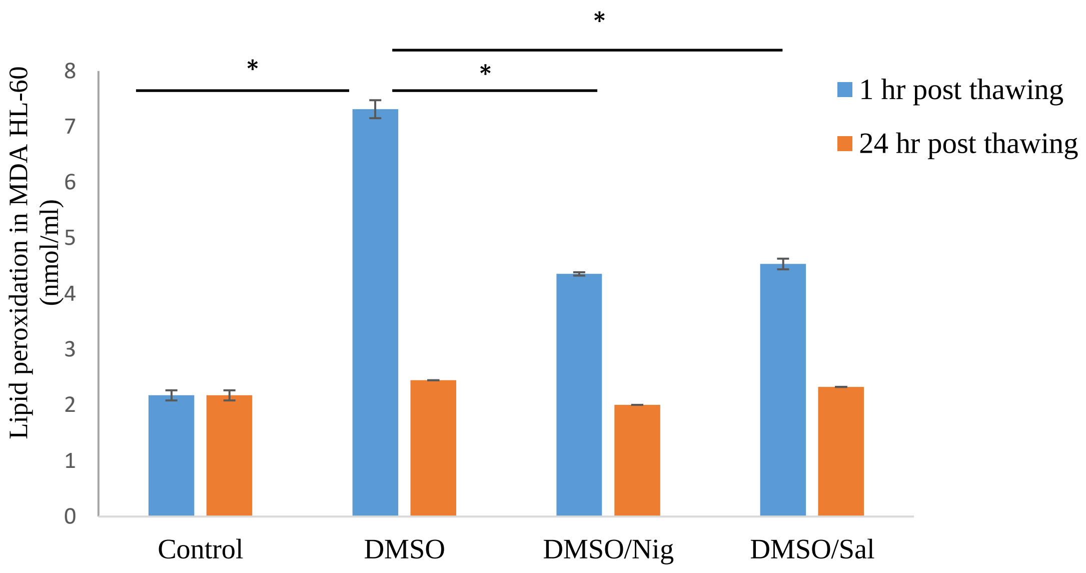

Click here to download Figure Figure 7 pdf..pdf

Figure 7.

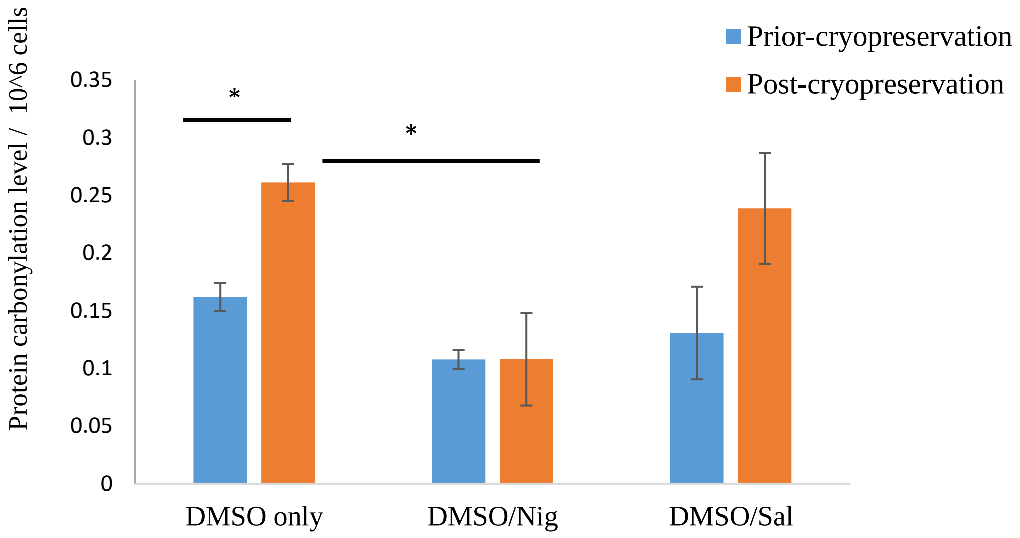

Click here to access/download
Supplementary Material
Supplement S1 pdf..pdf

Personal Cover
Click here to download Personal Cover Let. Giga Science Feb 2018.pdf

UNIVERSITY OF CAMBRIDGE
Chemical Engineering and Biotechnology

University of Cambridge
Chemical Engineering & Biotechnology Department
Philippa Fawcett Drive
Cambridge, CB3 0AS
UK

To:
Laurie Goodman, PhD
Editor-in-Chief
GigaScience

Thursday, 22 February 2018

Dear Laurie Goodman

We wish to submit our original manuscript entitled "**Human Leukaemia cells (HL-60) proteomic and biological signatures underpinning cryo-damage are differentially modulated by novel cryo-additives**" for your consideration. We confirm that this work is original and has not been published nor is it currently under consideration for publication elsewhere.

In this manuscript, we identified the proteomic profiles underpinning cryo-injury in DMSO with/without novel cryo-additive agents and validated the molecular profiles with the corresponding biological findings. Proteomic analysis and the corresponding functional readouts were combined to decipher, target and modulate cell-specific cryo-damaged pathways.

In summary Salidroside, and especially Nigerose were used as novel cryo-additive agents for the cryopreservation of HL-60 nucleated cells and have shown differential and/or an additive mode of action in comparison to the classically used DMSO by: i) modulating the level of protein expression associated with cryo-damage; ii) reducing proteins and/or lipid oxidation; ii) and increasing cell nuclear activities (DNA repair/duplication, RNA transcription and cell growth) and leading to an improved survival/proliferative rate post thaw.

The present data set could be of a major interest to your readers as such a discovery not only attempted to unravel and modulate the proteomic and biological profiles associated with cryo-damage but could potentially have a major impact on enhancing future cryo-media formulation. In addition, it opens up the possibility of using natural agents as cryo-additives (e. g. Nigerose or Salidroside) in stem cell, infertility treatment and potentially tissue engineering and regenerative medicine.

Thank you for your consideration and looking forward to hearing from you soon

Please address all correspondence concerning this manuscript to myself at hr288@cam.ac.uk

Sincerely,

Dr. Hassan Rahmoune
