# Supplementary material for: Human leukemia cells (HL-60) proteomic and biological signatures underpinning cryo-damage are differentially modulated by novel cryo-additives
Source: Gigascience. 2018 Dec 10;8(3):giy155. doi: 10.1093/gigascience/giy155 (PMC6394207; doi:10.1093/gigascience/giy155)
Supplement: Response_to_Reviewer_Comments_Original_Submission.pdf [file giy155_response_to_reviewer_comments_original_submission.pdf]

Author's Response To Reviewer Comments

Close

Dear Dr. Scott Edmunds,

Thank you for facilitating the review of our paper (Ref. GigaScience - GIGA-D-18-00064). You will find below that we agree with all of the reviewers' points, as indicated stepwise below. We have subsequently made the corresponding changes in the revised manuscript- See the 'Revised Manuscript with Track Changes' file.
We have also carried out a carful proof reading of our manuscript and amend it prior submitting the present revised version.

Kind regards,
Dr. Hassan Rahmoune

Comments to the Author Reviewer #1
Author response: we thank the referee for his/her comments and this has been altered accordingly as stated below:

So the authors should checked the following issues:
1)even if authors well summarized the changes that up to date have modified the cryopreservation technique, in the background section (line 70 pg 3), more detailed literature data on stem cell studies should be reported, considering their possible use in regenerative medicine;

Author response: The requested information has been included from line 70 up to line 78 and three references were also added. See the uploaded version entitled 'Revised Manuscript with Track Changes' file

2)at line 265 pg 10, for the sentence For example,……it's necessary delete the article OF; this should be checked;
Author response: we agree and the requested information has been included in line 314 in the uploaded version entitled 'Revised Manuscript with Track Changes' file

3) how many replicates for proteomic experiment were used ? The authors should specify it.
Author response: we have now added further information on this issue in the text lines 137, 706 and 713-714.
The requested information has also been included in Table 1, Tables S1, S2 and S3 legends. See the uploaded version entitled 'Revised Manuscript with Track Changes' file

4)Trypan blue assays for cell viability were carried out on HL-60 cell at 1 h, 24 h and 48 h post thaw. No description were reported in the results section, though in the Figure S1 for each novel cryo-agents cell viability rate are showed;

Author response: we agree and we have now added further information on this issue in the text on lines 243-245.
Cell viability was not reported here as HL-60 cells were washed centrifuged at 100 x g for 5 min and washed immediately three times with RPMI media (the changes in cell viability

post thaw was negligible (<2%).

5)also no cytotoxicity tests were performed; these results could strongly validate the proteome finding related to HL-60 cryopreservation in DMSO +/- Nig or Sal. The authors should consider this suggestion.
Author response: we thank the referee for his/her comments and this has been altered accordingly.
The lactate dehydrogenase assay was used as a measurements of cellular cytotoxicity and the requested information has been included in line 366-368 and the corresponding reference has also been added.

Reviewer #2:
1. I also miss tables that summarize the quantitative data, which is a must-have for quantitative proteomics manuscripts.
Author response: we thank the referee for his/her comments and this has been altered accordingly. The quantitative proteomics data analysis representing Arm 1, 2 & 3 were added as supplementary Tables S1, S2 and S 3, respectively.

2. Also, the authors out-of-a-sudden study carbonylation but do not give a rationale for this. To me it is unclear why they decided to look for this out of so many modifications.
Author response: this has been clarified and the requested information has been included on lines 39, 262, 272-274, 276, 278 and 732 (Figure 7 legend).

3. It is also unclear what the authors mean with "significantly quantified proteins", first I thought they meant significantly differential, however, the different numbers between the figures and table 1 make me wonder if I got that right. Thus, it is unclear what the total numbers of identified, quantified and significantly differentially expressed proteins are.

Author response: we agree and the requested information has been amended and included in lines 141-143 and 161-167.
The quantitative proteomics data representing Arm 1, 2 & 3 were added as a supplementary table S1, S2 and S 3 respectively. The biologically relevant proteome data summarised in table 1 now can be found in the supplementary tables S1, S2 and S3.

4. The discussion needs to be expanded, for instance addressing the question about the dynamic range of this study and the regulated proteome, if possible in the light of other studies on that cell line or at least compared to other cell lines.
5. Author response: we agree and the requested information has been amended and included in lines 305-308.

Some other issues:

1) The methods part is incomplete and important steps are not clear. The search algorithm is not clearly mentioned, it is also unclear what "default parameters for ion accounting" means. The authors should stick to standard guidelines for reporting proteomic MS data. The whole

part is a bit hard to follow, I wonder why the authors not report things step-by-step, which is first Progenesis alignment and peak detection, then export of peaklists and then a clearly described search strategy. Also it is unclear what the "Ion-matching requirements" mean, for instance 1 fragment per peptide and 3 fragments per protein. Why was O-GlcNac searched as PTM, this is not a common PTM one would include in the database search. On what level was the FDR, protein, peptide, PSM, all of them? Were all proteins that had an ANOVA below 0.05 considered as regulated, without an additional fold-change cut-off? I would expect high shares of false positives here. The authors should use a correctedp-value to compensate for that. Tables summarizing the quantitative data are missing as supplements.
Author response: we thank the referee for his/her comments and the missing tables summarizing the quantitative data representing Arm 1, 2 & 3 were added as Supplementary Table S1, S2 &S3 respectively.
The changes were also made accordingly in the text of the manuscript – See revised section “Proteomic analysis” – Page 6 and7.
We have also introduced the necessary changes in:
• “Sample preparation” section and introduced/updated the “data processing and database searches” – See page 16-18 in the revised version.
• The data processing and database searches sections were amended accordingly – See revised version of the manuscript: lines 448-469 (Page 17-18).

2) It is confusing to have the chapter on "data description" that contains incomplete information about database searches and quantification in the beginning of the manuscript and the actual part on M&M including the MS analysis in the end. The M&M part is not always clear. For instance what is "cooled acetone"?
Author response: we thank the referee for his/her comments and the "data description" section has been altered accordingly – See Line 449-469 (Page 17-18) in the revised version

3) Table 1: Fold changes are log2 I presume from looking at negative fold-changes, but it is not mentioned in the table. Or is -1.2 a 1.2-fold downregulation, which normally then would be 0.83?
Author response: we thank and agree with the referee for his/her comments and the table 1 have been amended in the revised version as the fold change is indeed a log2.

Close
