# Supplementary material for: Human leukemia cells (HL-60) proteomic and biological signatures underpinning cryo-damage are differentially modulated by novel cryo-additives
Source: Gigascience. 2018 Dec 10;8(3):giy155. doi: 10.1093/gigascience/giy155 (PMC6394207; doi:10.1093/gigascience/giy155)
Supplement: Response_to_Reviewer_Comments_Revision_1.pdf [file giy155_response_to_reviewer_comments_revision_1.pdf]

Author's Response To Reviewer Comments

Close

Dear Editor,
Please find attached the revised version of our manuscript, Ref "GIGA-D-18-00064R1, entitled: "Human Leukaemia cells (HL-60) proteomic and biological signatures underpinning cryo-damage are differentially modulated by novel cryo-additives". The referees and board member comments were addressed in the latest version Ref. "GIGA-D-18-00064R1.

On the behalf of the authors I would like to thank you, boards members and the reviewers for your collaboration.

Looking forward to hearing from you.

Kind regards

Dr, H.Rahmoune

Close
