# Supplementary material for: Human leukemia cells (HL-60) proteomic and biological signatures underpinning cryo-damage are differentially modulated by novel cryo-additives
Source: Gigascience. 2018 Dec 10;8(3):giy155. doi: 10.1093/gigascience/giy155 (PMC6394207; doi:10.1093/gigascience/giy155)
Supplement: Response_to_Reviewer_Comments_Revision_2.pdf [file giy155_response_to_reviewer_comments_revision_2.pdf]

Author's Response To Reviewer Comments

Close

Dear Sir/Madam

We have made the necessary corrections as suggested by Dr Scott Edmunds - See Version 26-11-2018.

Kind Regards

Dr Hassan Rahmoune

Close
