# Supplementary material for: Human leukemia cells (HL-60) proteomic and biological signatures underpinning cryo-damage are differentially modulated by novel cryo-additives
Source: Gigascience. 2018 Dec 10;8(3):giy155. doi: 10.1093/gigascience/giy155 (PMC6394207; doi:10.1093/gigascience/giy155)
Supplement: Reviewer_1_Report_(Original_Submission) -- Stefania Angelucci — 4/8/2018 Reviewed [file giy155_reviewer_1_report_(original_submission).pdf]

Reviewer Report

Title: Human Leukaemia cells (HL-60) proteomic and biological signatures underpinning cryo-damage are differentially modulated by novel cryo-additives

Version: Original Submission **Date:** 4/8/2018

Reviewer name: Stefania Angelucci

Reviewer Comments to Author:

This article discusses an interesting topic on cryopreservation methods to ensure long-term storage of viable cells; DMSO is the most commonly used cryoprotective agent (CPA), but it can induce cellular toxicity, change the cellular epigenetic state and produce patient-related side effects, so that today there is a growing demand for alternative molecules. It is well known that cryopreservative reagents (CPA) are required to reduce damage from cryopreservation. The authors evaluated the effects of new molecules on cryopreserved HL-60 cells such as nigerose or salidroside, combining them with cellular treatment with DMSO. The effects of these new CPAs and DMSO alone have been investigated with shotgun proteomic analysis by identifying key molecules and biochemical pathways related to CPA modulation of cryo-storage. I found this paper technically suitable and more importantly some data are look satisfactory. Even if this manuscript is acceptable for publication, many amendments are required to make this better.

So the authors should checked the following issues:

1)even if authors well summarized the changes that up to date have modified the cryopreservation technique, in the background section (line 70 pg 3), more detailed literature data on stem cell studies should be reported, considering their possible use in regenerative medicine;
2)at line 265 pg 10, for the sentence For example,……it's necessary delete the article OF; this should be checked;
3)how many replicates for proteomic experiment were used ? The authors should specify it.
4)Trypan blu assays for cell viability were carried out on HL-60 cell at 1 h, 24 h and 48 h post thaw. No description were reported in the results section, though in the Figure S1 for each novel cryo-agents cell viability rate are showed;
5)also no cytotoxicity tests were performed; these results could strongly validate the proteome finding related to HL-60 cryopreservation in DMSO +/- Nig or Sal. The authors should consider this suggestion.

Methods

Are the methods appropriate to the aims of the study, are they well described, and are necessary controls included? Yes

Conclusions

Are the conclusions adequately supported by the data shown? Yes

Reporting Standards

Does the manuscript adhere to the journal's guidelines on [minimum standards of reporting?](#) Yes

Choose an item.

Statistics

Are you able to assess all statistics in the manuscript, including the appropriateness of statistical tests used? Yes, and I have assessed the statistics in my report.

Quality of Written English

Please indicate the quality of language in the manuscript: Acceptable

Declaration of Competing Interests

Please complete a declaration of competing interests, considering the following questions:

- Have you in the past five years received reimbursements, fees, funding, or salary from an organisation that may in any way gain or lose financially from the publication of this manuscript, either now or in the future?
- Do you hold any stocks or shares in an organisation that may in any way gain or lose financially from the publication of this manuscript, either now or in the future?
- Do you hold or are you currently applying for any patents relating to the content of the manuscript?
- Have you received reimbursements, fees, funding, or salary from an organization that holds or has applied for patents relating to the content of the manuscript?
- Do you have any other financial competing interests?
- Do you have any non-financial competing interests in relation to this paper?

If you can answer no to all of the above, write 'I declare that I have no competing interests' below. If your reply is yes to any, please give details below.

I have no competing interest.

I agree to the open peer review policy of the journal. I understand that my name will be included on my report to the authors and, if the manuscript is accepted for publication, my named report including any attachments I upload will be posted on the website along with the authors' responses. I agree for my report to be made available under an Open Access Creative Commons CC-BY license (http://creativecommons.org/licenses/by/4.0/). I understand that any comments which I do not wish to be included in my named report can be included as confidential comments to the editors, which will not be published.

I agree to the open peer review policy of the journal

To further support our reviewers, we have joined with Publons, where you can gain additional credit to further highlight your hard work (see: https://publons.com/journal/530/gigascience). On publication of this paper, your review will be automatically added to Publons, you can then choose whether or not to claim your Publons credit. I understand this statement.

Yes Choose an item.
