# Supplementary material for: Human leukemia cells (HL-60) proteomic and biological signatures underpinning cryo-damage are differentially modulated by novel cryo-additives
Source: Gigascience. 2018 Dec 10;8(3):giy155. doi: 10.1093/gigascience/giy155 (PMC6394207; doi:10.1093/gigascience/giy155)
Supplement: Reviewer_2_Report_(Original_Submission) -- Rene Zahedi — 4/27/2018 Reviewed [file giy155_reviewer_2_report_(original_submission).pdf]

Reviewer Report

Title: **Human Leukaemia cells (HL-60) proteomic and biological signatures underpinning cryo-damage are differentially modulated by novel cryo-additives**

Version: **Original Submission** **Date:** 4/27/2018

Reviewer name: **Rene Zahedi**

Reviewer Comments to Author:

Al-Otaibi et al. describe the proteomic analysis of HL-60 cells upon inflicted cyro-damage. Although of general interest, I cannot recommend the manuscript for publication at the current state. The authors have to considerably improve the presentation of their data, including the figures and interpretation of their results. I also miss tables that summarize the quantitative data, which is a must-have for quantitative proteomics manuscripts. Also, the authors out-of-a-sudden study carbonylation but do not give a rationale for this. To me it is unclear why they decided to look for this out of so many modifications. It is also unclear what the authors mean with "significantly quantified proteins", first I thought they meant significantly differential, however, the different numbers between the figures and table 1 make me wonder if I got that right. Thus, it is unclear what the total numbers of identified, quantified and significantly differentially expressed proteins are. The discussion needs to be expanded, for instance addressing the question about the dynamic range of this study and the regulated proteome, if possible in the light of other studies on that cell line or at least compared to other cell lines.

Some other issues:

1) The methods part is incomplete and important steps are not clear. The search algorithm is not clearly mentioned, it is also unclear what "default parameters for ion accounting" means. The authors should stick to standard guidelines for reporting proteomic MS data. The whole part is a bit hard to follow, I wonder why the authors not report things step-by-step, which is first Progenesis alignment and peak detection, then export of peaklists and then a clearly described search strategy. Also it is unclear what the "Ion-matching requirements" mean, for instance 1 fragment per peptide and 3 fragments per protein. Why was O-GlcNac searched as PTM, this is not a common PTM one would include in the database search. On what level was the FDR, protein, peptide, PSM, all of them? Were all proteins that had an ANOVA below 0.05 considered as regulated, without an additional fold-change cut-off? I would expect high shares of false positives here. The authors should use a corrected p-value to compensate for that. Tables summarizing the quantitative data are missing as supplements.
2) It is confusing to have the chapter on "data description" that contains incomplete information about database searches and quantification in the beginning of the manuscript and the actual part on M&M including the MS analysis in the end. The M&M part is not always clear. For instance what is "cooled acetone"?
3) Table 1: Fold changes are log2 I presume from looking at negative fold-changes, but it is not mentioned in the table. Or is -1.2 a 1.2-fold downregulation, which normally then would be 0.83?

Methods

Are the methods appropriate to the aims of the study, are they well described, and are necessary controls included? Choose an item.

Conclusions

Are the conclusions adequately supported by the data shown? Yes

Reporting Standards

Does the manuscript adhere to the journal's guidelines on [minimum standards of reporting?]() Choose an item.

Choose an item.

Statistics

Are you able to assess all statistics in the manuscript, including the appropriateness of statistical tests used? Choose an item.

Quality of Written English

Please indicate the quality of language in the manuscript: Choose an item.

Declaration of Competing Interests

Please complete a declaration of competing interests, considering the following questions:

- Have you in the past five years received reimbursements, fees, funding, or salary from an organisation that may in any way gain or lose financially from the publication of this manuscript, either now or in the future?
- Do you hold any stocks or shares in an organisation that may in any way gain or lose financially from the publication of this manuscript, either now or in the future?
- Do you hold or are you currently applying for any patents relating to the content of the manuscript?
- Have you received reimbursements, fees, funding, or salary from an organization that holds or has applied for patents relating to the content of the manuscript?
- Do you have any other financial competing interests?
- Do you have any non-financial competing interests in relation to this paper?

If you can answer no to all of the above, write 'I declare that I have no competing interests' below. If your reply is yes to any, please give details below.

I declare that I have no competing interests

I agree to the open peer review policy of the journal. I understand that my name will be included on my report to the authors and, if the manuscript is accepted for publication, my named report including any attachments I upload will be posted on the website along with the authors' responses. I agree for my report to be made available under an Open Access Creative Commons CC-BY license (http://creativecommons.org/licenses/by/4.0/). I understand that any comments which I do not wish to be included in my named report can be included as confidential comments to the editors, which will not be published.

I agree to the open peer review policy of the journal

To further support our reviewers, we have joined with Publons, where you can gain additional credit to further highlight your hard work (see: https://publons.com/journal/530/gigascience). On publication of this paper, your review will be automatically added to Publons, you can then choose whether or not to claim your Publons credit. I understand this statement.

Yes Choose an item.
